# An Integrative Multiomics Framework for Identification of Therapeutic Targets in Pulmonary Fibrosis

**DOI:** 10.1002/advs.202207454

**Published:** 2023-04-10

**Authors:** Muhammad Arif, Abhishek Basu, Kaelin M. Wolf, Joshua K. Park, Lenny Pommerolle, Madeline Behee, Bernadette R. Gochuico, Resat Cinar

**Affiliations:** ^1^ Section on Fibrotic Disorders National Institute on Alcohol Abuse and Alcoholism National Institutes of Health Rockville MD 20852 USA; ^2^ Laboratory of Cardiovascular Physiology and Tissue Injury National Institute on Alcohol Abuse and Alcoholism National Institutes of Health Rockville MD 20852 USA; ^3^ Laboratory of Physiologic Studies National Institute on Alcohol Abuse and Alcoholism National Institutes of Health Rockville MD 20852 USA; ^4^ Medical Genetics Branch National Human Genome Research Institute National Institutes of Health (NIH) Bethesda MD 20892 USA

**Keywords:** bleomycin, CB_1_R, IPF, Irf5, metabolomics, mouse model, multi‐omics, network biology, pulmonary fibrosis, systems biology, systems pharmacology, transcriptomics

## Abstract

Pulmonary fibrosis (PF) is a heterogeneous disease with a poor prognosis. Therefore, identifying additional therapeutic modalities is required to improve outcome. However, the lack of biomarkers of disease progression hampers the preclinical to clinical translational process. Here, this work assesses and identifies progressive alterations in pulmonary function, transcriptomics, and metabolomics in the mouse lung at 7, 14, 21, and 28 days after a single dose of oropharyngeal bleomycin. By integrating multi‐omics data, this work identifies two central gene subnetworks associated with multiple critical pathological changes in transcriptomics and metabolomics as well as pulmonary function. This work presents a multi‐omics‐based framework to establish a translational link between the bleomycin‐induced PF model in mice and human idiopathic pulmonary fibrosis to identify druggable targets and test therapeutic candidates. This work also indicates peripheral cannabinoid receptor 1 (CB_1_R) antagonism as a rational therapeutic target for clinical translation in PF. Mouse Lung Fibrosis Atlas can be accessed freely at https://niaaa.nih.gov/mouselungfibrosisatlas.

## Graphical abstract

Multi‐omics approach helps to establish a translational link in idiopathic pulmonary fibrosis (IPF) between human and its experimental model in mice. Multi‐omics‐based framework assists the identification of druggable targets in IPF. Systems pharmacology endorses cannabinoid CB_1_R antagonism as a rational therapeutic strategy in IPF.

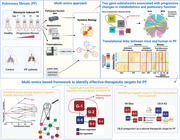

## 1 Introduction

Pulmonary fibrosis (PF) is a progressive and heterogeneous interstitial lung disease associated with a poor prognosis. Although there are two approved medications to treat pulmonary fibrosis, there is still a need to identify effective therapeutic modalities.^[^ ^1^, ^2^ ^]^ Lack of predictive and progressive disease biomarkers in the clinic, aside from pulmonary function testing, represents a significant gap in the preclinical to clinical translational process. Furthermore, most of the preclinically validated effective anti‐fibrotic drug candidates were not found to be efficacious in clinical trials, suggesting missing links between the animal model and human disease.^[^ ^2^ *^–^* ^4^ ^]^ Importantly, this gap also poses a challenge in designing rational combination therapies in idiopathic pulmonary fibrosis (IPF), despite evidence suggesting the promise of multi‐targeted therapy in treating fibrotic disorders.^[^ ^2^ ^]^ Currently, bleomycin‐induced PF in mice is a commonly used preclinical model for IPF despite its limitations, such as severe initial acute inflammation followed by progressive fibrosis compared to the chronic progressive course of IPF in patients.^[^ ^5^, ^6^ ^]^ Importantly, clinically approved medications, such as pirfenidone and nintedanib, proved their therapeutic efficacy in PF using the bleomycin‐induced PF model.^[^ ^7^, ^8^ ^]^ which demonstrated its value as the current gold standard preclinical model for testing therapeutic candidates for PF. This model for PF is also recognized by the American Thoracic Society.^[^ ^5^ ^]^ However, we should establish a translational bridge between the bleomycin‐induced PF model and its clinical relevance to make this model more effective in identifying therapeutic targets and designing rational combination therapies. Eventually, this could facilitate translational strategies from the bench to the clinic for PF treatment.

**Figure 1:**
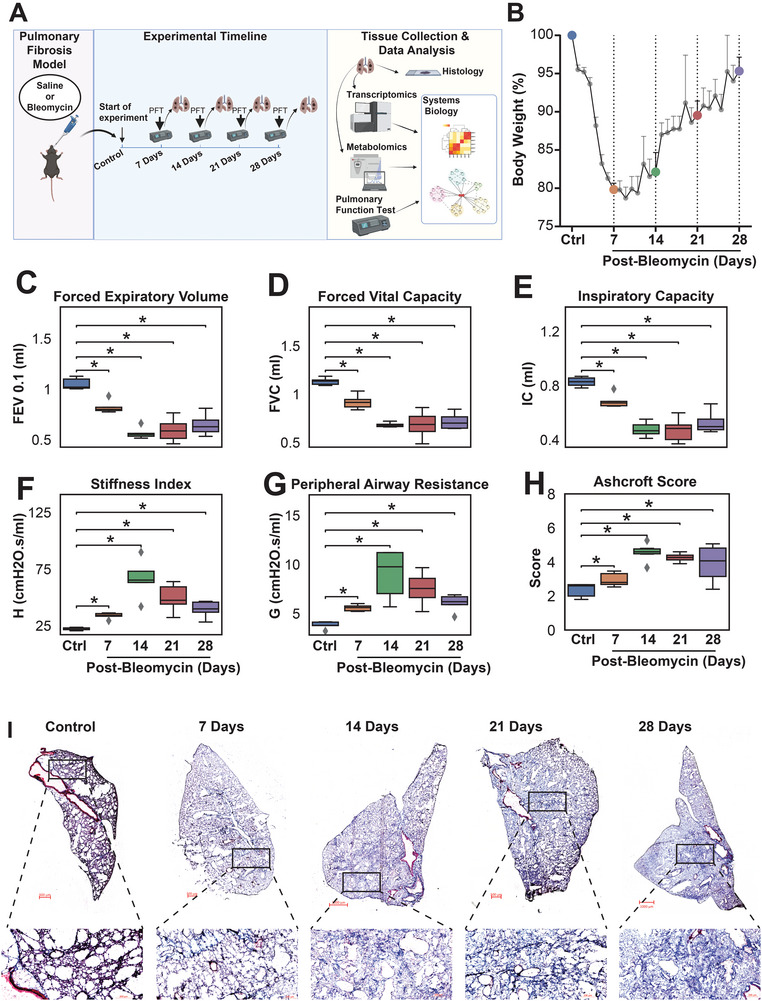
Study design and model validations. A) Experimental design. B) Body weight change of the mouse after a single dose of oropharyngeal bleomycin (1 U kg^−1^) administration (*n* = 5 per group). C–G) Pulmonary Function Test (PFT) parameters forced expiratory volume 0.1 s (FEV_0.1_), forced vital capacity (FVC), and inspiratory capacity (IC), stiffness index and peripheral airway resistance in all measured time points after the administration of bleomycin when compared to control (*n* = 5 per group; one‐way ANOVA; *: *p*‐value < 0.05). H) Ashcroft Score in all measured time points after the administration of bleomycin when compared to control. I) Masson's Trichrome staining of the mouse lung in day 0 (control), and 7‐, 14‐, 21‐, and 28‐days after the bleomycin administration.

Most of our biochemical and genomics understanding of PF in humans is based on the late presentation of disease. Therefore, the animal model could also be instrumental to understand the progressive nature and the critical pathologic pathways in the early, intermediate, and late stages of the disease. Eventually, these may require a systems‐level understanding of disease initiation and progression in bleomycin‐induced PF to maximize translational value in the clinic.^[^ ^9^, ^10^ ^]^ To address this, we assessed and identified progressive alterations in pulmonary function, histopathology, transcriptome, and metabolome in mice lungs at 7, 14, 21, and 28 days after a single dose of oropharyngeal (OP) bleomycin (1 U kg^−1^) (**Figure** **1**A). Using co‐expression network analysis, we identified two gene subnetworks, G‐1 and G‐2, having a central role in multiple changes in the transcriptome and metabolome as well as pulmonary function. To establish the translational relevance of the animal model, we demonstrated similarities in the transcriptomic landscape of fibrotic murine lungs at 14 days post‐bleomycin compared to lung biopsy samples from IPF patients. Interestingly, the two identified gene subnetworks and their relevant biological pathways in mice also exist in the lungs of IPF patients, which helped to establish clinical relevance. To identify common regulators of the multiple pathological pathways, we performed transcriptional network analysis that revealed gene tracks T‐1 and T‐2 out of 13 tracks as major regulators of G‐1 and G‐2 in the gene co‐expression network (GCN). We hypothesized that an ideal therapeutic candidate for PF should be able to attenuate fibrotic changes in the G‐1 and G‐2 subnetworks of GCN. Then, we demonstrated that cannabinoid receptor 1 (CB_1_R) overactivation could contribute to the PF pathology by three subnetworks out of five in GCN, G‐0, G‐1, and G‐2, which are the major drivers of the transcriptomics changes in PF. Our study not only endorsed the therapeutic potential of peripheral CB_1_R antagonism for clinical translation but also demonstrated the benefit of using the multi‐omics‐based framework to identify druggable targets for pharmacological intervention and developing rational combination therapies using the bleomycin‐induced PF model.

## 2 Results

### 2.1 Progressive Decline in Pulmonary Function and Development of Fibrosis in Bleomycin‐Induced PF in Mice

To establish a progressive disease framework in the experimental model of PF with clinical relevance, we generated multi‐omics data (transcriptomics and metabolomics) from lung tissue and pulmonary function tests (PFTs) in bleomycin‐induced PF at 7, 14, 21, and 28 days after a single dose of OP bleomycin (Figure 1A). Bleomycin administration induced ≈20% body weight loss due to tissue injury and inflammation within the first 14 days, in agreement with previous observations (Figure 1B).^[^ ^6^ ^]^ PFTs and high‐resolution computed tomography (HRCT) imaging of the lungs are used by clinicians to determine the progression of PF in clinical practice, and forced vital capacity (FVC), a PFT parameter, is currently the only marker acknowledged by regulatory authorities to evaluate therapeutic efficacy of investigational treatments in limiting the progression of PF. Therefore, we measured PFT parameters using Flexivent in bleomycin‐induced PF to determine the rate of decline in pulmonary function in mice. PFT parameters, such as forced vital capacity (FVC), forced expiratory volume (FEV), inspiratory capacity (IC), stiffness index (H), and peripheral airway resistance (G) declined significantly until 14 days after bleomycin, and remained stable thereafter (Figure 1C–G, Table S1, Supporting Information). In line with a decline in pulmonary function, collagen deposition also increased progressively and peaked at 14 days as observed by Masson's trichrome staining and Ashcroft scoring (Figure 1H,I). As expected, bleomycin also increased gene expression of fibrogenic markers, collagen type 1 alpha 1 (*Col1a1*), fibronectin 1 (*Fn1*), TIMP metallopeptidase inhibitor 1 (*Timp1*), and vimentin (*Vim*) (Figure S1, Supporting Information).

### 2.2 Progressive Changes in Transcriptomics in Lungs in Bleomycin‐Induced PF

**Figure 2:**
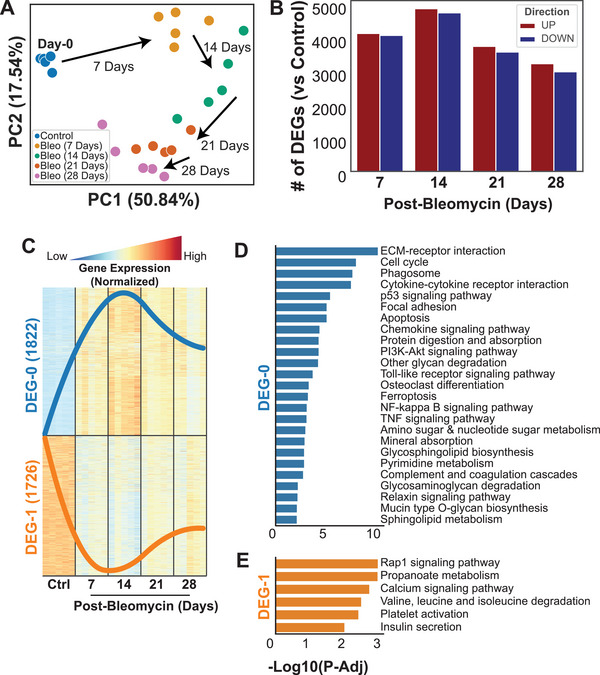
Transcriptomics data analysis. A) Principal Component Analysis (PCA) showed each time point from mice at day 0 (control), and 7‐, 14‐, 21‐, and 28‐days after the bleomycin administration (*n* = 5 per group). B) The number of differentially expressed genes (DEGs, FDR < 0.05) showed significant transcriptional differences between control and post‐bleomycin mice. C) The trend of normalized expressions of genes that were differentially expressed in all four time points compared to control showed that, even for the commonly dysregulated genes, the magnitudes of up‐ and down‐regulation (DEG‐0 and DEG‐1, respectively) were peaking in 14 days post‐bleomycin compared to control. D,E) Significantly associated pathways (FDR < 0.05) with genes in DEG‐0 and DEG‐1 of the commonly dysregulated genes in all time points.

Principal component analysis (PCA) from the bulk transcriptomics data showed a time‐dependent progressive shift in the lung transcriptome after bleomycin (**Figure** **2**A). This shift could be attributed to a progressive change as it shifted in the same direction at each time point (Figure 2A). Between 3000 to 5000 genes were significantly differentially expressed, being either up‐ or downregulated at each time point (Figure 2B, Table S1, Supporting Information). The highest number of differentially expressed genes (DEGs) were observed at 14 days after bleomycin, where 4730 genes were upregulated and 4854 genes were downregulated (Figure 2B, Table S1, Supporting Information). Furthermore, we identified 1822 upregulated DEGs (designated as DEG‐0) and 1726 downregulated DEGs (DEG‐1), which were consistent at all time points compared to control lungs (Figure 2C, Figure S2, Supporting Information). In both DEG‐0 and DEG‐1, the most significant changes were observed at 14 days post‐bleomycin (Figure 2C, Figure S2, Supporting Information). As the progressive decline in pulmonary function and development of fibrosis were evident at each time point, we wanted to identify biological pathways concordant with these changes throughout disease progression. Therefore, we investigated biological pathways associated with DEG‐0 and DEG‐1, where we observed 25 upregulated pathways associated with DEG‐0 (Figure 2D) and six downregulated pathways associated with DEG‐1 (Figure 2E). The top six upregulated pathways were related to extracellular matrix (ECM)‐receptor interaction, cell cycle, phagosome, cytokine–cytokine receptor interaction, p53 signaling, and focal adhesion. The top six downregulated pathways were related to Rap1 signaling, propanoate metabolism, calcium signaling, valine, leucine, and isoleucine degradation (branched‐chain amino acids, BCAA), platelet activation, and insulin secretion (Figure 2E).

### 2.3 GCN Analysis Revealed Two Subnetworks That Centrally Interact with All the Other Subnetworks in the Transcriptome of Fibrotic Lungs in Mice

**Figure 3:**
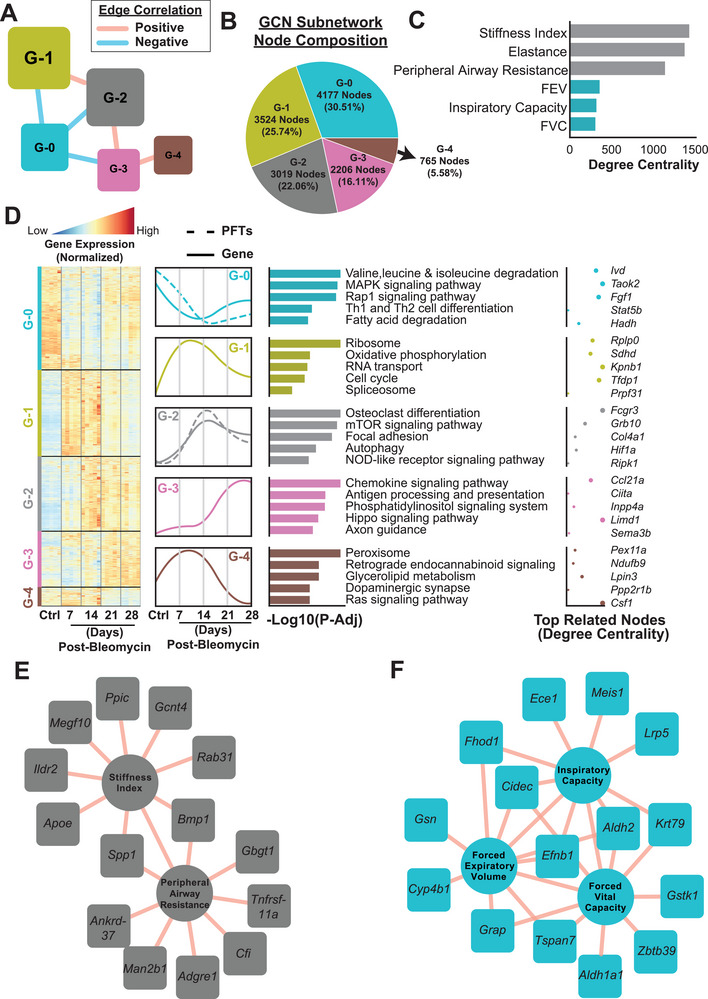
Gene Co‐Expression Network (GCN) analysis. A) Five subnetworks with distinct network architectures were detected in the GCN with node size proportional to their average local transitivity in the network (numbers in the bracket denoted the total number of genes and PFTs in each subnetwork). B) The node composition of GCN subnetworks. C) Centrality analysis of PFTs. Stiffness index, elastance, and peripheral airway resistance were the most central PFTs connected to more than 1000 genes in the GCN, while other PFTs (FEV, inspiratory capacity, and FVC) were connected to less than 400 genes. D) The detailed view of each subnetwork, including the normalized gene expression trends in each sample, the median trajectory of the gene and PFTs in control and four post‐bleomycin time points, associated KEGG pathways to the subnetworks, and the genes with the highest degree related to the KEGG pathways. E) The top 10 highest correlated GCN nodes to stiffness index and peripheral airway resistance. F) Forced vital capacity, forced expiratory volume, and inspiratory capacity.

To study the functional relationship of genes and identify critical pathways associated with the pathology of PF progression, we generated a PF progression GCN and selected highly connected genes (top 25% positively correlated gene pairs with a false‐discovery rate <0.05) (Table S2, Supporting Information). To better understand the network structure, we performed Leiden community detection^[^ ^11^ ^]^ and identified five subnetworks in the GCN (Table S2, Supporting Information): G‐0 (4177 nodes), G‐1 (3524 nodes), G‐2 (3019 nodes), G‐3 (2206 nodes), and G‐4 (765 nodes) (**Figure** **3**A,B). G‐1 and G‐2 were defined as central subnetworks (highest average clustering coefficient)^[^ ^12^ ^]^ and interacting closely with all other subnetworks (Figure 3A). G‐1 and G‐2 were also consisting of 47.80% of the nodes in the GCN (Figure 3B). To explore the functional relationships between the decline in pulmonary functions and the transcriptional changes in the GCN, we integrated PFT data into the GCN and performed centrality analysis with PFT parameters (Figure 3C, Table S2, Supporting Information). Stiffness index, elastance, and peripheral airway resistance were the most central PFT parameters, and their alteration patterns were connected to 1432; 1377; and 1143 genes, respectively (Figure 3B, Table S2, Supporting Information). On the other hand, FEV, IC, and FVC were connected to 363, 325, and 32 genes in the GCN (Figure 3C, Table S2, Supporting Information). Detailed view and gene expression trends of the subnetworks showed that the G‐0 subnetwork, which includes genes associated with pathways, such as BCAA degradation, MAPK signaling, Rap1 signaling, Th1 and Th2 cell differentiation, and fatty acid degradation, was significantly downregulated throughout all time points, with the lowest expression level at day 14 post‐bleomycin (Figure 3D). Genes in G‐1 subnetwork, which are associated with pathways such as ribosome, oxidative phosphorylation, RNA transport, cell cycle, and spliceosome, were upregulated, peaking at day 7 and 14 post‐bleomycin; thereafter, it showed a recovery trend at 21‐ and 28‐days post‐bleomycin (Figure 3D). G‐2 subnetwork comprised genes associated with osteoclast differentiation, mTOR signaling pathway, focal adhesion, autophagy, and NOD‐like receptor signaling pathways. This subnetwork progressively increased until 14 days post‐bleomycin, then showed a recovery trend but remained upregulated at a steady level at 21 and 28 days. The genes in G‐3 had slightly higher expression at day 7 compared to control, increased significantly at day 21, and remained elevated at day 28 (Figure 3D). Those genes are associated with chemokine signaling, antigen processing and presentation, phosphatidylinositol signaling, Hippo signaling and axon guidance (Figure 3D). Finally, genes in G‐4 subnetwork showed unique trends, increasing significantly at day 7 and then decreasing at day 21 and 28 compared to control (Figure 3D). This subnetwork was composed of genes belonging to peroxisome, glycerolipid metabolism, dopaminergic synapse, and Ras signaling pathways. Interestingly, pulmonary function parameters, such as stiffness index, elastance, and peripheral airway resistance, were clustered with genes in G‐2 subnetwork progressive trend, whereas FEV, FVC, and IC were clustered with G‐0 subnetwork (Figure 3A–C, Table S2, Supporting Information). The top 10 highest correlated genes in GCN to stiffness index and peripheral airway resistance included secreted phosphoprotein 1 (*Spp1*) and bone morphogenic protein 1 (*Bmp1*), which were correlated with both PFT parameters (Figure 3E). The top 10 highest correlated genes in GCN to FEV, FVC and IC included aldehyde dehydrogenase 2 (*Aldh2*), and ephrin beta 1 (*Efnb1*), which were correlated with all the three PFT parameters (Figure 3F).

### 2.4 Progressive Changes of the Lung Metabolome in Bleomycin‐Induced PF in Mice

**Figure 4:**
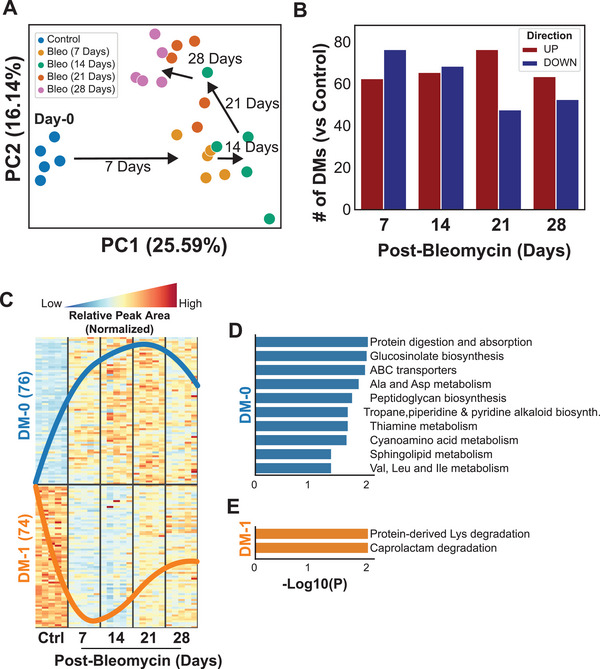
Metabolomics data analysis. A) Principal Component Analysis (PCA) showed each time point from mice at day 0 (control), and 7‐, 14‐, 21‐, and 28‐days after the bleomycin administration (*n* = 5 per group). B) The number of differential metabolites (DMs, FDR < 0.05) showed significant metabolic differences between control and post‐bleomycin mice. C) The trend of normalized relative peak area of metabolites that were significantly different in at least two time points compared to control showed that most up‐regulated metabolites (DM‐0) were peaking on 21 days and down‐regulated metabolites (DM‐1) on 7 days post bleomycin. D,E) Significantly associated pathways (*p*‐value < 0.05) with metabolites in DM‐0 and DM‐1 of the commonly dysregulated genes in at least two time points.

In addition to the transcriptomics changes in fibrotic lungs, we also examined dysregulations in metabolomics from the same subjects in mice. Using an untargeted metabolomics approach, we quantified 459 metabolites, but 137 of those were removed due to a high rate of missing values (Table S3, Supporting Information). As observed in the transcriptomics (Figure 2A), PCA also demonstrated a significant time‐dependent shift in metabolomics in the lungs after bleomycin (**Figure** **4**A). Significant change was observed 7 days after bleomycin compared to control, then it progressed time dependently (Figure 4A). A range of 40–80 metabolites detected either increased or decreased significantly at each time point compared to the control (Figure 4B, Table S3, Supporting Information). We found 76 metabolites significantly upregulated in at least two time points and defined as DM‐0 (Figure 4C), and 74 metabolites were significantly downregulated in at least two time points and defined as DM‐1 (Figure 4C). The trend of normalized relative peak area of metabolites demonstrated that most significant changes in metabolomics occurred at days 7 and 14 and peaked at day 21 after bleomycin for the upregulated metabolites in DM‐0, whereas the downregulated metabolites in DM‐1 were at their lowest level at 7 and 14 days (Figure 4C). Ten pathways associated with differentiated metabolome were upregulated in DM‐0 (Figure 4D), whereas two pathways were downregulated, which were associated with DM‐1 (Figure 4E). The most significantly upregulated six pathways were related to protein digestion and absorption, glucosinoleate biosynthesis, ABC transporters, alanine and aspartate metabolism, peptidoglycan biosynthesis, tropane, piperidine, and pyridine alkaloid biosynthesis (Figure 4D). In contrast, downregulated pathways were related to protein‐derived lysine degradation and caprolactam degradation (Figure 4E).

### 2.5 Integration of GCN and Metabolite Correlation Network (MCN) Revealed that Metabolic Subsystems and MCN Subnetworks are Connected to Main GCN Subnetworks

**Figure 5:**
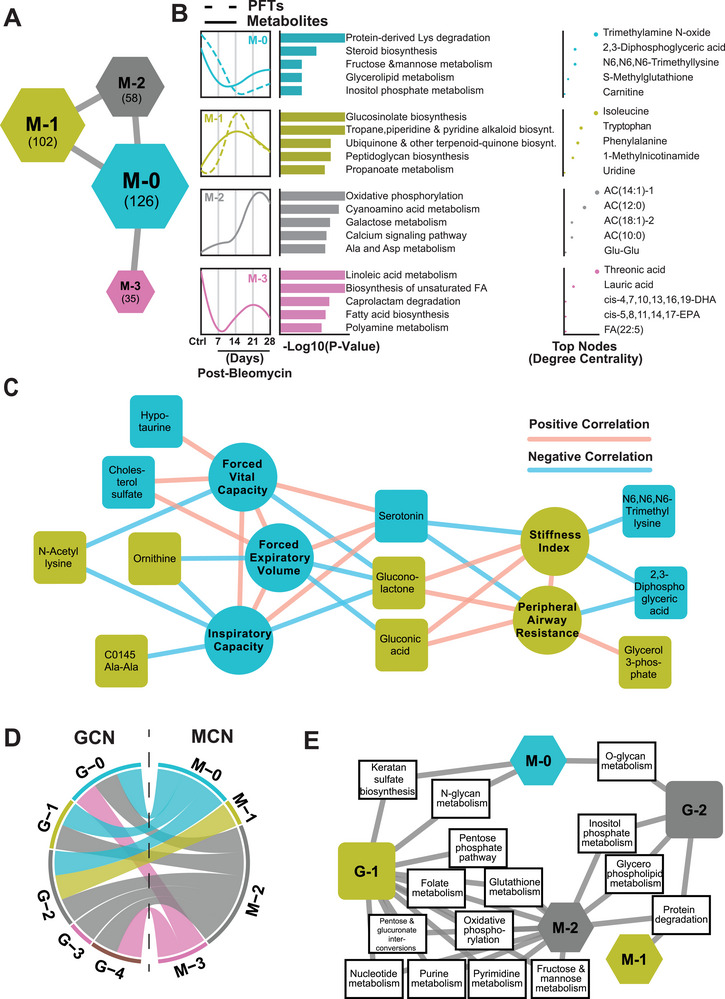
Metabolite Correlation Network (MCN) analysis. A) Four subnetworks with distinct network architectures were detected in the MCN with node size proportional to their average local transitivity in the network (numbers in the bracket denoted the total number of metabolites and PFTs in each subnetwork). B) The detailed view of each subnetwork, including the median trajectory of the metabolites and PFTs in control and four post‐bleomycin time points, associated metabolic pathways, and top metabolite nodes, based on the degree centrality, of each subnetwork. C) The top 10 highest correlated MCN nodes to pulmonary functions. D) GCN and MCN clusters were integrated based on their metabolic subsystems based on mouse metabolic models retrieved from https://metabolicatlas.org. E) Metabolic subsystems connecting two main GCN clusters (G‐1 and G‐2) to the MCN clusters.

We identified four metabolic subnetworks as M‐0 (126 metabolites), M‐1 (102 metabolites), M‐2 (58 metabolites), and M‐3 (35 metabolites), using MCN analysis (**Figure** **5**A, Table S4, Supporting Information). M‐0 subnetwork metabolites were decreased significantly at all time points compared to control, with the lowest level at day 14. These metabolites are associated with pathways such as protein‐derived lysine degradation, steroid biosynthesis, fructose and mannose metabolism, glycerolipid metabolism, and inositol phosphate metabolism (Figure 5B). M‐1 subnetwork metabolites were progressively increased, showing a peak at day 14 post‐bleomycin, then showed a recovery trend but remained at high levels at days 21 and 28 (Figure 5B). M‐1 subnetwork metabolites are associated with pathways such as glucosinoleate biosynthesis, tropane, piperidine and pyridine alkaloid biosynthesis, ubiquinone and other terpen‐quinone biosynthesis, peptidoglycan biosynthesis, and propanoate metabolism (Figure 5B). M‐2 subnetwork metabolite levels slightly increased at day 7, significantly elevated at day 21, and remained elevated at day 28 (Figure 5B). The M‐2 subnetwork metabolites are involved in pathways such as oxidative phosphorylation, cyanoamino acid metabolism, galactose metabolism, calcium signaling, alanine, and aspartate metabolism (Figure 5B). M‐3 subnetwork metabolites were significantly decreased at day 7 then showed progressive recovery trend until day 21, which remained at low levels, and then declined again at day 28 (Figure 5B). M‐3 metabolites are associated with pathways such as linoleic acid metabolism, biosynthesis of unsaturated fatty acids, caprolactam degradation, fatty acid biosynthesis, and polyamine metabolism (Figure 5B). M‐0 has the highest average clustering coefficient, thus being the most central subnetwork, and it interacts with all other subnetworks (Figure 5A). Furthermore, we observed that the median trajectory of the metabolites in M‐0 and M‐1 subnetworks showed similar time‐dependent trend with pulmonary function test parameters (Figure 5B). The top 10 metabolites in MCN mostly correlated with pulmonary function parameters and included gluconolactone, gluconic acid, and serotonin, which were highly correlated with all five PFT parameters (FVC, FEV, IC, stiffness index, and peripheral airway resistance) (Figure. 5C).

A combination of M‐0 and M‐1 represents the trends of 228 metabolites, that is, 71% of total metabolites detected in the lungs from bleomycin‐induced PF. These results suggest that metabolites within the M‐0 and M‐1 subnetworks and associated pathways might have critical roles in fibroproliferative changes and pulmonary function. To understand the functional interaction between the transcriptomics and the metabolomics data in lungs with bleomycin‐induced PF, we integrated GCN and MCN subnetworks based on their metabolic subsystems using the mouse metabolic models (https://metabolicatlas.org) (Figure 5D). We found that metabolic subsystems associated with metabolites from M‐0, M‐1, and M‐2 MCN subnetworks, which consist of 89% of total metabolites, were connected to the two main GCN subnetworks (G‐1 and G‐2) (Figure 5D, Figure S3, Supporting Information). G‐1 subnetwork was connected to M‐0 with N‐glycan metabolism and keratan sulfate biosynthesis, and to M‐2 with oxidative phosphorylation, folate metabolism, glutathione metabolism, pentose phosphate pathway, pentose glucuronate interconversions, fructose and mannose metabolism, and purine and pyrimidine metabolism (Figure 5E). G‐2 subnetwork was connected to M‐0 with O‐glycan metabolism, to M‐1 with protein degradation, and to M‐2 with inositol phosphate metabolism and glycerophospholipid metabolism (Figure 5E).

### 2.6 Central Driver Genes From the GCN in Fibrotic Lungs in Mice Provides Translational Link to Human IPF Patients

**Figure 6:**
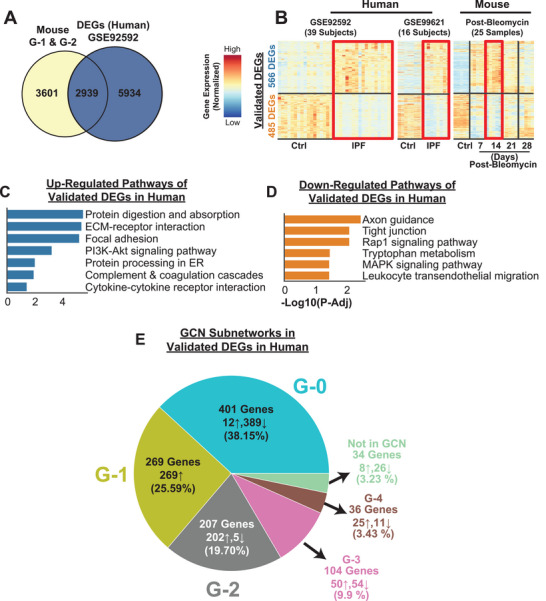
Translational link in fibrotic lung transcriptome between human IPF patients and bleomycin‐induced PF in mice A) 2,939 genes (≈45%) from two main GCN clusters (G‐1 and G‐2) were significantly differentially expressed in late‐stage human IPF patients based on an independent cohort (GSE92592) B) Validation with two independent human IPF cohorts (55 Subjects) showed high transcriptional profile similarities (1051 validated genes) between human IPF patients and 14 days post‐bleomycin mice. C,D) Significant KEGG pathways (FDR < 0.05) associated with the up‐ and down‐regulated genes validated in 2 independent human cohorts. E) The composition of GCN subnetworks in the validated DEGs in two late‐stage human IPF patients’ cohorts (6B). More than 45% of the genes belong to the two main GCN clusters (G‐1 and G‐2).

Our findings suggest that the top central G‐1 and G‐2 subnetworks of the GCN might be a major regulator of the pathological changes in the transcriptome (Figure 3A), metabolome (Figure 5D,E), and pulmonary function in bleomycin‐induced PF. In recent years, multiple studies with bulk and single‐cell RNA sequencing from the lungs of IPF patients and their respective controls have been reported and made publicly available. The time course of acute lung injury followed by progressive fibrosis in the bleomycin‐induced PF model is well characterized, and we focused here on comparing the transcriptomic changes of fibrotic lungs from mice versus those in humans to bridge gaps in understanding between the preclinical model and clinical patients and to uncover the fibroproliferative pathways conserved in both species. Since we identified G‐1 and G‐2 subnetworks as having a central role interacting with all other subnetworks in mice, one of the outstanding questions is whether the genes in the G‐1 and G‐2 subnetworks exist and are regulated similarly in fibrotic lungs of IPF patients. Answering this question would help establish a translational link between the preclinical mouse model and clinical presentation of IPF, as well as to identify effective therapeutic targets. To explore this, we employed two different cohorts of lung transcriptomics data sets from control and IPF patients [GSE92592^[^ ^13^ ^]^ and GSE99621^[^ ^14^ ^]^]. First, using one of the largest cohorts from Schafer et al., which contains 19 control and 20 IPF patients, we found that there was a significant overlap with the G‐1 and G‐2 subnetworks (**Figure** **6**A, Table S5, Supporting Information) in fibrotic lungs between mice and human. G‐1 and G‐2 subnetworks of GCN have 6540 genes in mice, 45%^[^ ^2^ ^]^ of them were also differentially expressed in IPF patients’ lungs as well (Figure 6A, and 1431 genes in GSE99621 human IPF patient cohort, Figure S4A, Supporting Information), suggesting translational relevance.

### 2.7 Transcriptomic Signature from Fibrotic Lungs at Day 14 Post‐Bleomycin in Mice Resembles IPF Patients’ Lung

One of the major gaps between the human PF and bleomycin‐induced PF is the time resolution. This raises an important question: which time point or time points in bleomycin‐induced PF best resembles the transcriptomic signature of human PF. Comparing two different cohorts of transcriptomics in IPF patients’ lung and bleomycin‐induced fibrotic lungs, we found that 14 days post‐bleomycin resembled the transcriptional changes that were observed in late‐onset IPF patients’ lung (Figure 6B, Table S5, Supporting Information). Comparing DEGs in G‐1 and G‐2 subnetworks at 14 days with the two human cohorts, we found that 68.81% and 59.96% of DEGs were regulated with same trend in GSE92592 and GSE99621, respectively (Figure S4B,C, Supporting Information). Considering the heterogeneity of PF, we focused on the DEGs and biological pathways, which are regulated in same trends in all cohorts. In total, we observed 1051 genes that showed the same trends in bleomycin‐induced PF and both independent human IPF cohorts (Figure 6B). Similar transcriptomics landscape and DEGs in both IPF patients and fibrotic lungs from mice also contain genes and pathways from the G‐1 and G‐2 subnetworks (Figure 6B,C). Accordingly, the relevant pathways, such as protein digestion and absorption, ECM‐receptor interaction, focal adhesion, PI3K‐Akt signaling, protein processing in endoplasmic reticulum, complement and coagulation cascades, and cytokine–cytokine receptor interaction were upregulated in the lungs of IPF patients (Figure 6C). In contrast, axon guidance, tight junction, Rap1 signaling, tryptophan metabolism, MAPK signaling, and leukocyte transendothelial migration pathways, were downregulated in the lungs of IPF patients (Figure 6D). Furthermore, we checked the GCN subnetworks composition in the validated DEGs in both human late‐stage IPF patient cohorts (Figure 6B) and found that 475 DEGs (45.29%) belong to the two main GCN clusters, G‐1 and G‐2 (Figure 6E, Figure S4D,E, Supporting Information, for single human cohort validations). Since G‐1 and G‐2 subnetwork composition is 47.8% of GCN in the fibrotic lungs in mice (Figure 3B), this further exhibits the translatability of the GCN main clusters, G‐1 and G‐2, in human IPF patients’ lung.

### 2.8 Identifying Transcriptional Factors Regulating Critical Fibroproliferative Changes in the Lungs

**Figure 7:**
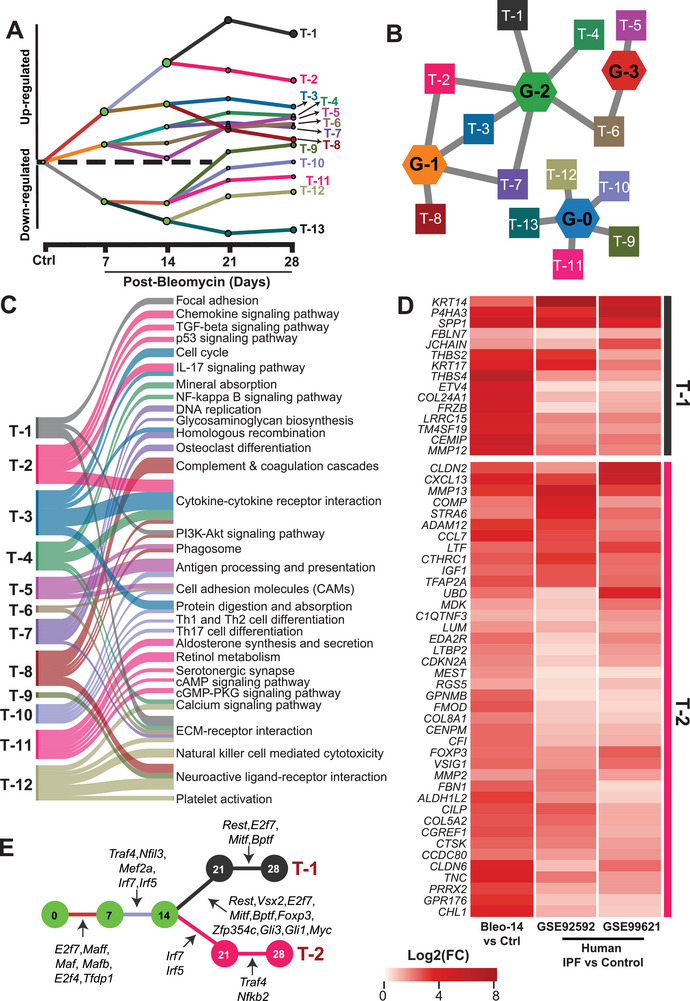
Identifying common transcriptional regulators of critical fibroproliferative pathways. A) Predicted transcriptional regulation models from the iDREM analysis showed 13 gene tracks with distinct trajectories. Each dot annotated the junction where transcription factors and their regulated gene expressions diverge. B) The association of GCN subnetworks to the identified gene tracks. C) Top five enriched KEGG Pathways for each gene track. D) 55 validated T‐1 and T‐2 target genes in fibrotic lungs transcriptome shared in both human IPF patients’ cohorts and bleomycin‐challenged mice. E) The transcription factors that regulated the progression of genes in tracks T‐1 and T‐2 at each time point. T‐1 and T‐2 were associated with important functions related to pulmonary fibrosis, inflammation, and associated specifically with the two most central GCN subnetworks (G‐1 and G‐2).

One of the potential approaches in regulating strongly associated gene networks would be finding their common regulator such as transcription factors. Therefore, we were interested in whether we could cluster networks and associated pathways based on their transcription factors common to those subnetworks, which may be targeted for therapeutic intervention to regulate multiple critical pathologic pathways at once. In this regard, we assessed the regulation of transcription factors at different time points of PF progression in the GCN using transcriptional regulation models with interactive dynamic regulatory events miner (iDREM) analysis.^[^ ^15^ ^]^ Although transcriptional regulator activity of transcription factors may not be well reflected by their mRNA expression levels.^[^ ^16^, ^17^ ^]^ transcription factors and iDREM analysis could help us with identifying and clustering gene networks under the control of those particular transcriptional factors. Accordingly, the predicted transcriptional regulation model demonstrated 13 gene tracks with distinct trajectories, which were marked from T‐1 to T‐13 (**Figure** **7**A, Table S6, Supporting Information). We aimed to identify certain tracks which belonged to G‐1 and G‐2 subnetworks, since they were the central subnetworks for the GCN. Seven out of 13 gene tracks were enriched with genes from G‐1 and G‐2 subnetworks and designated as T‐1, T‐2, T‐3, T‐4, T‐6, T‐7, and T‐8 (Figure 7B). Then, we further explored the top five enriched pathways and biological processes for each gene track to understand their function (Figure 7C, Figure S5, and Table S6, Supporting Information). Importantly, we focused on the most significantly upregulated or downregulated tracks, which were showing a progressive trend and remained significantly altered throughout the time course of PF (Figure 7A). Track T‐1 and T‐2 were the most significantly upregulated gene tracks and connected to G‐1 and G‐2 subnetworks (Figure 7B). Track T‐1 was associated with focal adhesion, PI3K‐Akt signaling, and ECM‐receptor interaction pathways (Figure 7C, Table S6, Supporting Information). Track T‐2 was associated with chemokine signaling, TGF*β* signaling, p53 signaling, IL‐17 signaling, and cytokine–cytokine receptor interaction pathways (Figure 7C, Table S6, Supporting Information). Interestingly, all pathways associated with T‐1 and T‐2 were implicated in inflammation, fibrosis, and epithelial‐mesenchymal transition, which suggests that T‐1 and T‐2 might be particularly critical gene tracks for the development and progression of PF. Additionally, 55 genes with in the T‐1 and T‐2 gene tracks similarly and significantly upregulated in fibrotic lungs of mice and two independent human IPF cohorts (Figure 7D).Furthermore, we note that the clinical significance of these pathways associated with T‐1 and T2 have previously been described.^[^ ^18^ *^–^* ^21^ ^]^ Furthermore, some of these pathways, such as PI3K‐Akt signaling pathways^[^ ^22^ ^]^ and ECM‐receptor interaction,^[^ ^23^ ^]^ have been studied in clinical trials, providing successful outcomes at certain endpoints. Accordingly, pharmacological modulation of T‐1 and T‐2 gene tracks within G‐1 and G‐2 subnetworks might be an attractive therapeutic strategy for PF. Importantly, T‐1 and T‐2 tracks were regulated by the same transcription factors until 14 days post‐bleomycin (Figure 7E, Table S6, Supporting Information), at which point, we observed a peak decline in pulmonary function and increase fibrosis suggesting fibrosis onset in lungs (Figure 1B,H), and also, resembling human IPF transcriptomics signature (Figure 6B). The transcription factors identified for T‐1 and T‐2 are as follows: for day 7, *E2f4*, *E2f7*, *Maff*, *Maf*, *Mafb*, and *Tfdp1*; and for day 14: *Irf5*, *Irf7*, *Traf4*, *Nfil3*, and *Mef2a* (Figure 7E, Table S5, Supporting Information).

### 2.9 IRF5 and IRF7 are Critical Transcription Factors Regulating top Central G1 and G2 Subnetworks of the GCN in Mouse and Human PF

Based on our findings, we hypothesized that targeting G‐1 and G‐2 transcriptional networks in relation to the transcriptional regulation of T‐1 and T‐2 tracks might be critical to attenuate disease progression and to develop effective therapies. According to this hypothesis, one could expect that attenuation of the transcription factors activity prevents and/or attenuates the development of PF by regulating multiple downstream pathogenic pathways. We identified *Irf5*, *Irf7*, *Traf4*, *Nfil3*, and *Mef2a* as transcriptional regulators of T‐1 and T‐2 tracks activated on days 7 and 14 (Figure 7E). We focused on track T‐2 as it interacted with both central GCN subnetworks, G‐1 and G‐2. IRF5 and IRF7 were also transcription factors for track T‐2 between days 14–21, which resembles the late onset of the disease. In this case, preventing the activation of IRF5 and/or IRF7 may have therapeutic benefit in inhibiting the development of PF and/or attenuating progression of PF. In human, bulk transcriptomics data derived from IPF patients’ lungs represent late‐stage PF since they were obtained from explanted lung tissue, therefore they may be lacking well separated time resolution in regard to disease progression. Besides, some of the transcription factors may be expressed predominantly in certain cell types such as IRF5 in immune cells.^[^ ^24^ ^]^ Therefore, bulk transcriptomics also lacks spatial resolution especially in human lungs with heterogeneity of sampling location. Importantly, mRNA expression of transcriptional factors may not represent its activity^[^ ^16^, ^17^ ^]^ Considering all these points, one approach to assess importance of their activity in the pathology is to find evidence from epidemiologic studies in human and/or loss of function studies in animals. Indeed, there is evidence that IRF5 deletion prevents the development of fibrosis in experimental models of liver^[^ ^25^ ^]^ skin, and lung fibrosis.^[^ ^26^ ^]^ In addition, IRF7 deletion also prevented the development of skin and lung fibrosis.^[^ ^27^ ^]^ The pathologic role of both IRF5 and IRF7 has been demonstrated in human pathology as well,^[^ ^25^ *^–^* ^27^ ^]^ with activating IRF5 polymorphism serving as a predictive genetic marker for the predisposition of scleroderma‐related organ fibrosis.^[^ ^26^ ^]^ Additionally, the activation status of the pathways in the gene tracks may indicate activation status of their transcriptional factors identified by iDREM analysis.^[^ ^15^, ^28^ ^]^ In this study, IRF5 and IRF7 have also emerged as transcriptional factors of fibrogenic pathways in T‐2 gene track such as TGF*β* signaling, p53 signaling, chemokine signaling, cytokine–chemokine receptor interaction, and IL‐17 signaling (Figure 7C). Align with the lung transcriptomics in mice (Figure 7C), the pathways and genes in‐T‐1 and T‐2 gene tracks were also increased similarly in IPF patient's lung transcriptome (Figures 6B,C and 7D). This evidence suggests IRF5 and IRF7 as emerging therapeutic targets for PF treatment and demonstrate the translational value of our multi‐omic framework. Although regulating transcription factors could efficiently regulate multiple critical fibrogenic pathways as shown in the framework here, it may not be viable to directly target them for drug development, considering their pleiotropic roles in physiologic homeostasis, as well as pathology. Therefore, an ideal approach would be identifying an upstream druggable target for selectively regulating the transcription factor of interest in the pathological state. Accordingly, an ideal drug candidate for PF would be an upstream regulator of IRF5 and/or IRF7 to attenuate the fibrosis‐mediated activation of the gene networks such as T‐1 and T‐2.

How we can use the presented framework to identify druggable targets: Deletion of CB_1_R attenuated bleomycin‐induced alteration in G‐0, G‐1, and G‐2 subnetworks in the GCN

To exemplify the use of the presented framework in target identification for PF, we tested whether CB_1_R might be a therapeutic target for PF treatment. We chose CB_1_R because we previously showed that genetic deletion of CB_1_R attenuates bleomycin‐induced increase in IRF5 expression as well as fibrosis.^[^ ^29^ ^]^ Also, IRF5 is a crucial downstream mediator of diabetogenic CB_1_R signaling that triggers cytokine release in pro‐inflammatory macrophages.^[^ ^30^ ^]^ Furthermore, CB_1_R belongs to G‐2 subnetwork (Table S2, Supporting Information). Accordingly, we hypothesized that antifibrotic mechanism of CB_1_R in PF might be regulating multiple pathological pathways within the G‐1 and G‐2 transcriptomics networks.

**Figure 8:**
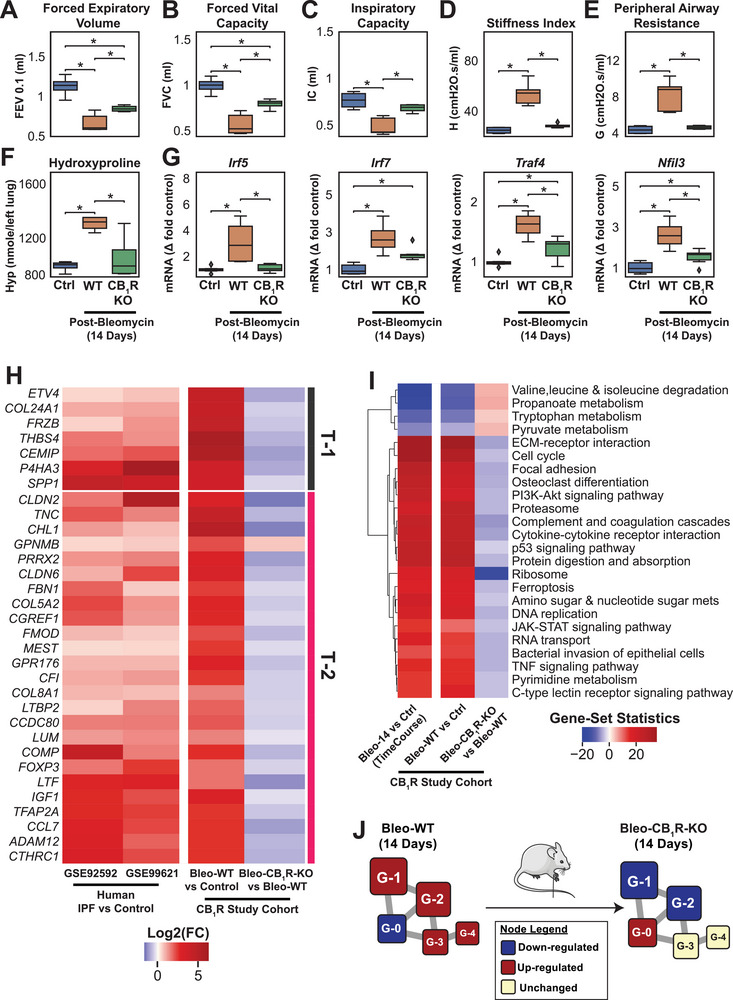
Identifying cannabinoid receptor 1 (CB_1_R) antagonism as a rational therapeutic target in pulmonary fibrosis. A–E) Deletion of CB_1_R prevented bleomycin‐induced decline in pulmonary function parameters (stiffness index, peripheral airway resistance, inspiratory capacity [IC], forced expiratory volume 0.1 s [FEV_0.1_], and forced vital capacity [FVC]; *n* = 4 for Ctrl and CB_1_R KO, and *n* = 5 for WT; one‐way ANOVA; *: *p*‐value < 0.05). F) Hydroxyproline level decreased significantly in CB_1_R KO mice compared to wild‐type after the administration of bleomycin (*n* = 4 for Ctrl and CB_1_R KO, and *n* = 5 for WT; one‐way ANOVA; *: *p*‐value < 0.05). G) Gene expression of *Irf5*, *Irf7*, *Traf4*, and *Nfil3*, transcriptional regulators of T‐1 and T‐2 gene tracks (*n* = 6 for Ctrl and CB_1_R KO, and *n* = 4 for WT; one‐way ANOVA; *: *p*‐value < 0.05). H) PF‐induced 31 validated T‐1 and T‐2 target genes in both human and mice were significantly attenuated with the deletion of CB_1_R compared to wild‐type mice at 14 days post bleomycin. (*p*‐value < 0.05) I) The majority of important pulmonary fibrosis‐related KEGG pathways, such as ECM‐receptor interaction, PI3K‐Akt signaling, focal adhesion, amino‐acid metabolism, and immune and inflammatory pathways, were significantly attenuated with the deletion of CB_1_R compared to wild‐type mice at 14 days post bleomycin. J) The deletion of CB_1_R in the lung reversed the dysregulation of genes 14 days post‐bleomycin in the three biggest GCN subnetworks: G‐0 (up‐regulated), G‐1 (down), and G‐2 (down), while G‐3 and G‐4 remained unchanged, compared to wild‐type mouse 14 days post‐bleomycin.

While 14‐days post‐bleomycin transcriptomic signature in lungs resembled human IPF patients’ lung, we characterized the effect of the deletion of CB_1_R on the GCN alterations by using CB_1_R^+/+^ wildtype (WT) and CB_1_R^−/−^ (CB_1_R KO) mice at 14 days post‐bleomycin time point. We found that the deletion of CB_1_R improved pulmonary function parameters (**Figure** **8**A–E, Table S7, Supporting Information) and attenuated the bleomycin‐induced fibrosis as quantified by hydroxyproline (Figure 8F), which is in agreement with previous observations.^[^ ^29^ ^]^ Deletion of CB_1_R also attenuated bleomycin induced gene expression of *Irf5*, *Irf7*, TNF*α*‐receptor activated factor 4 (*Traf4)*, Nuclear factor interleukin 3 regulated (*Nfil3)* (Figure 8G) but not *Mef2a* (Figure S6, Supporting Information), which are the transcriptional factors of T‐1 and T‐2 gene tracks (Figure 7E). Importantly, deletion of CB_1_R attenuated fibrosis induced expression of 32 genes within the T‐1 and T‐2 tracks (Figure 8H), which are the activated genes in the lungs of IPF patients from two independent cohorts (Figures 7D and 8H). Indeed, among these genes such as cell migration induced hyaluronidase 1 (*CEMIP*),^[^ ^31^ ^]^ osteopontin (*SPP1*),^[^ ^32^ ^]^ prolyl 4‐hydroxylase subunit alpha 3 *(P4HA3*),^[^ ^33^ ^]^ tenascin C (*TNC*),^[^ ^34^ ^]^ transmembrane glycoprotein NMB *(GPNMB)*,^[^ ^35^ ^]^ insulin‐like growth factor 1 *(IGF1)*,^[^ ^36^ ^]^ cartilage oligomeric matrix protein (*COMP*),^[^ ^37^ ^]^ ADAM metallopeptidase domain 12 (*ADAM12*),^[^ ^38^ ^]^ collagen triple helix repeat containing 1 (*CTHRC1)*,^[^ ^39^ ^]^ claudin 2 (*CLDN2*),^[^ ^40^ ^]^ latent transforming growth factor beta binding protein 2 (*LTBP2*)^[^ ^41^ ^]^ were proven to involve disease pathology and correlated with disease severity in IPF patients. Accordingly, this further reinstate potential pathologic role of CB_1_R in IPF. Overall, deletion of CB_1_R also attenuated multiple bleomycin‐induced transcriptional changes compared to the WT mice (Figure 8I). Our data demonstrated that CB_1_R in lungs involves the regulation of 24 pathways including ECM‐receptor interaction, focal adhesion kinase, osteoclast differentiation, PI3K‐Akt signaling, cytokine–cytokine receptor interaction, JAK‐STAT signaling, and TNF signaling (Figure 8I). Most importantly, the deletion of CB_1_R downregulated bleomycin‐induced upregulation of G‐1 and G‐2 subnetworks, while upregulating bleomycin‐induced downregulation of the G‐0 subnetwork (Figure 8J). These results suggest the potential therapeutic effect of CB_1_R antagonism by regulating multiple critical pathways in three subnetworks, G‐0, G‐1, and G‐2, out of five subnetworks in the GCN of PF.

## 3 Discussion

Here we presented a multi‐omic framework for bridging translational gaps between the common animal model of PF and human IPF to facilitate target identification and prioritization for IPF drug discovery. We integrated transcriptomics, metabolomics, and pulmonary function parameters to compare with temporal resolution of PF‐associated alterations between mouse and human pathology. Then, we identified major drivers of GCN, as well as their interaction with pulmonary function and metabolomic features. We also provided evidence that transcriptomic signatures of fibrotic lungs in mice at 14 days post‐bleomycin overlaps with human lung fibrosis. Most importantly, we identified central gene subnetworks such as G‐1 and G‐2 from GCN (Figure 3, Table S2, Supporting Information) and found that the genes and the identified pathways also exist in the fibrotic lung transcriptome from IPF patients, which reconfirmed the translational value of the framework (Figure 6A, Table S5, Supporting Information). 89% of the differential metabolites belonging to the M‐0, M‐1, and M‐2 of the MCN were connected to G‐1 and G‐2 subnetworks (Figure 5A,D). Accordingly, based on this framework, targeting G‐1 and G‐2 subnetworks could attenuate pathological transcriptional pathways, metabolic dysregulation, and decline in pulmonary function in PF. To regulate multiple pathways targeting G‐1 and G‐2 subnetworks, we identified IRF‐5 and IRF‐7 as the common transcription factors of gene track T‐2 (Figure 7E), which interacted with both G‐1 and G‐2 subnetworks (Figure 7B) and regulating pathways of TGF*β* signaling, chemokine signaling, p53 signaling, IL‐17 signaling, cytokine–cytokine receptor interaction (Figure 7C). Furthermore, we presented a proof‐of‐concept example that deletion of CB_1_R, an upstream regulator of IRF5,^[^ ^29^, ^30^ ^]^ attenuated pathological changes in G‐0, G‐1, and G‐2 subnetworks in the GCN of fibrotic lung transcriptome, underscoring its potential as an emerging therapeutic target for PF treatment (Figures 8A–E and 7C).

In the bleomycin‐induced PF model, the most dynamic changes in PFT parameters, transcriptome, and metabolome were observed at days 7 and 14 after bleomycin administration, suggesting fibrosis establishment and progression within the first 14 days. Therefore, day 7 may represent the early disease onset; meanwhile, days 14 and 21 could represent the late onset of the disease,^[^ ^42^, ^43^ ^]^ which aligns with previous observations. Indeed, human IPF patients’ transcriptomic data also resembled the transcriptomic landscape on day 14 post‐bleomycin, those human subjects representing the late disease onset since the lung tissues derived from lung transplant patients. Furthermore, in PCA plots at 14 days’ time point in both transcriptomics (Figure 2A) and metabolomics (Figure 4A) showed more variation in data compared to the other time points, and transitional trend between days 7 and 21, which suggests heterogeneous progression that aligns with the heterogeneity observed in disease progression in human IPF.^[^ ^44^ ^]^ Our findings may provide rationale to start any preclinical therapeutic intervention between days 7 and 21, as a model for starting treatment at the early disease stage and between days 14 and 28 for testing therapeutic efficacy at later disease stages. Another practical use of our study is that preclinical efficacy testing studies can be designed in a target‐specific manner using GCN and assessing the target gene interaction with regulating pathological changes at a certain time point.

One of the major issues in the clinic for IPF is the lack of predictive and progressive markers to understand the functional relationship between pulmonary function and other biological metrics in fibrotic lungs. Currently, pulmonary function testing is the only clinical outcome to assess disease progression and therapeutic efficacy of potential drug candidates in clinical trials. To identify the interaction between pulmonary function with other ‐omic parameters in disease progression, we integrated 6 PFT parameters acquired by Flexivent with transcriptomic and metabolomic parameters in mice to conduct unsupervised analysis. Through centrality analysis, we found that the stiffness index has the most centrality to interact and correlate with 1432 genes in the G‐2 subnetwork (Figure 3C) of the GCN and 85 metabolites in the M‐1 subnetwork of the MCN (Table S4, Supporting Information). One of the top ten genes highly correlated with both stiffness index and peripheral airway resistance was *Spp1*, which encodes osteopontin (Figure 3E). Fibrosis promoting role of high osteopontin expression was shown in IPF patients.^[^ ^32^ ^]^ Later, high *SPP1*‐expressing macrophages are identified as contributing to IPF.^[^ ^45^ ^]^ Additionally, *SPP1*‐expressing myofibroblasts have also been emphasized as a fibrogenic myofibroblast population in IPF patients’ lungs.^[^ ^39^ ^]^ In agreement with the previous observations, we also showed an increased expression of *SPP1* in fibrotic lungs in human and mice (Figure 7D). In a recent study, silencing *Spp1* attenuated bleomycin‐induced PF.^[^ ^46^ ^]^ These multiple studies have thus endorsed the pathologic significance of *SPP1* overexpression in IPF. Indeed, our finding demonstrated its strong association with pulmonary function decline in fibrotic lungs in bleomycin‐induced PF (Figure 3E). Furthermore, deletion of CB_1_R attenuated bleomycin induced expression of *Spp1* in bleomycin‐induced PF (Figure 8H), suggesting regulatory of CB_1_R on osteopontin. IPF is a restrictive lung disease characterized by progressive decline in several PFT parameters, including FVC, FEV_1_, total lung capacity (TLC), and diffusion capacity (DLCO), and PFTs are used to monitor patients with IPF. In fibrotic murine lungs, FVC and FEV interacted with about 363 and 312 genes, respectively, in the G‐0 subnetwork of the GCN (Figure 3C) and with 72 and 79 metabolites, respectively, in M‐0 subnetwork of the MCN (Table S4, Supporting Information). Recently, it was shown that *Aldh2* gene expression was significantly reduced in activated myofibroblasts in fibrotic lungs in bleomycin‐induced PF,^[^ ^47^, ^48^ ^]^ accordingly *Aldh2* was identified as a critical gene for myofibroblast programming in PF. This agrees with the current finding that one of the top ten genes highly correlated with the decline in FVC, FEV, and IC was *Aldh2* (Figure 3E). In metabolomics, we found that the levels of gluconolactone and gluconic acid were significantly increased and positively correlated with stiffness index and peripheral airway resistance whereas negatively correlated with FVC and FEV parameters. Importantly, both gluconolactone and gluconic acid belong to the pentose phosphate pathway (Figure 5C). This result suggests that the pentose phosphate pathway is upregulated in bleomycin‐induced PF, which is highly correlated with a decline in pulmonary function. Indeed, it was previously demonstrated that the pentose phosphate pathway was the most significantly increased pathway in PF.^[^ ^49^ ^]^ In addition, pharmacometabolomic evidence has shown that pirfenidone treatment does not reverse the altered pentose phosphate pathway in PF.^[^ ^49^ ^]^ Considering the strong relationship between the decline in pulmonary function and the activation of the pentose phosphate pathway, it would be important to identify a potential therapeutic candidate for PF which attenuates pentose phosphate pathway in PF. Identifying such a therapeutic candidate could be a complementary approach to pirfenidone treatment. Overall, the current study could also establish a relationship in alterations between pulmonary function parameters with the lung transcriptome and metabolome.

Considering the PCA in both transcriptomics and metabolomics in fibrotic murine lungs, there was a dramatic shift at day 7 post bleomycin in the metabolome, which did not further shift at day 14 (Figure 4A,C). On the other hand, there was a significant, progressive alteration between days 7 and 14 in the transcriptome (Figure 2A,C). This suggests that there is a robust change in the lung metabolome at early disease stages not seen in the lung transcriptome. In IPF, it is essential to identify the patients at the early disease stage to start antifibrotic therapy as early as possible to maximize therapeutic benefit. Accordingly, identifying clinically validated biomarkers in the early disease stage is very critical. In future metabolomics studies in lungs, bronchoalveolar lavage fluid (BALF), and blood might be quite instrumental and important for potential biomarker studies especially integrating multi‐omics approaches with lung transcriptomics, proteomics, and pulmonary function parameters.

In recent years, the transcriptomics approach has been widely used in fibrotic lungs from IPF patients, which was instrumental to uncover the critical role of certain genes in identifying them as potential therapeutic targets. However, PF is a polygenic and multifactorial disease. Therefore, it would be better to identify critical pathways and their regulation with the disease progression as GCN. Then, assessing GCN correction from the pathological state to the homeostatic state with therapeutic intervention would be a potential approach to develop effective therapeutic strategies. One of the critical limitations associated with focusing on a gene that could have central, pleiotropic functions in physiology and pathophysiology would be observing adverse events due to the inhibition of critical pathways for physiological function. For instance, the PI3K‐Akt pathway is identified as one of the major pathways in PF from our analysis both in mice and human as being in G‐1 subnetwork (Figures 6C and 7C). Indeed, the role of PI3K was well described earlier in preclinical and clinical studies.^[^ ^20^, ^50^ ^]^ Even successful clinical trials were conducted with a PI3K inhibitor (omipalisib) with a positive effect on the efficacy endpoint.^[^ ^22^ ^]^ However, the clinical trial was terminated due to adverse effects.^[^ ^22^ ^]^ This clinical trial outcome exemplified that despite the evident efficacy, directly targeting PI3K pathway for inhibition is not a viable strategy since PI3K has pathological as well as important physiological functions. A better strategy is to find druggable therapeutic targets which could be an upstream regulator of the PI3K pathway in the pathological state, responsible for its overactivation. Targeting such a regulator could provide therapeutic efficacy without adverse effects. Here, we exemplified that CB_1_R deletion not only attenuated the PI3K pathway but also attenuated multiple pathologic pathways including focal adhesion kinase, ECM‐receptor interaction, cytokine–cytokine receptor interaction with reversing fibrotic changes in G‐0, G‐1, and G‐2 subnetworks in GCN. Deletion of CB_1_R reversed alteration of three subnetworks out of five including G‐1 and G‐2, which are the most central subnetworks of the GCN in mice as well as exist in human IPF. Endocannabinoid AEA is a bioactive lipid and an endogenous agonist of CB_1_Rs. Its increase in tissues along with CB_1_R expression indicates overactivity of CB_1_Rs.^[^ ^51^, ^52^, ^53^ ^]^ AEA levels significantly increased in the BALF of IPF and Hermansky‐Pudlak syndrome‐pulmonary fibrosis (HPSPF) patients.^[^ ^29^, ^54^ ^]^ In addition, CB_1_R protein expressions were also dramatically increased in fibrotic lungs from IPF and HPSPF patients.^[^ ^29^, ^54^ ^]^ Importantly, increased AEA level in BALF was negatively correlated with PFT parameters such as FVC, TLC, and DLCO in both IPF and HPSPF.^[^ ^29^, ^54^ ^]^ All these demonstrated that overactivity of CB_1_R contributed to the disease progression in IPF and HPSPF. Then, pharmacological antagonism of CB_1_R provided therapeutic benefits attenuating progression in bleomycin‐induced PF.^[^ ^29^, ^54^ ^]^ Accordingly, CB_1_R antagonism was identified as a potential therapeutic target for both IPF and HPSPF.^[^ ^29^, ^54^ ^]^ First‐generation CB_1_R antagonist rimonabant was used in the clinic for obesity and metabolic disorders. There was no observed cardiovascular or systemic toxicity and adverse effects other than neuropsychiatric side effects due to the blockade of CB_1_R in the central nervous system (CNS).^[^ ^55^ ^]^ Recently introduced second and third generation peripherally restricted CB_1_R antagonists could avoid CNS mediated adverse effects.^[^ ^55^ ^]^ This could make peripheral CB_1_R antagonism an emerging therapeutic strategy for PF, as well as other fibrotic and metabolic disorders.^[^ ^55^ ^]^ To date, peripheral CB_1_R antagonists have not been tested in clinical trials for PF, but considering previous^[^ ^29^, ^54^ ^]^ and current evidence from this study, the potential therapeutic benefit of CB_1_R antagonism in PF remains to be tested with clinically available peripheral CB_1_R antagonists such as MRI‐1867 (zevaquenabant) and MRI‐1891.

Considering the multi‐factorial nature of pulmonary fibrosis, effective therapy would be possible with multi‐target approach such as combination therapy or polypharmacology to cure PF. Combination therapy clinical trials are challenging due to regulatory perspective and drug–drug interaction related concerns. Furthermore, IPF is a rare disease, and the statistical power of the study brings an additional challenge for clinical trials with combination therapy for IPF. Therefore, selecting a rational combination therapy is even more important for rare diseases like PF. We identified five transcriptomic subnetworks of GCN. In an ideal rational combination therapy, all the pathological changes in all subnetworks should be reversed back to physiological state. Based on the presented framework, any potential therapeutic candidate for PF should be regulating the top central subnetworks such as G‐1 and G‐2, then the other drug could target probably the remaining subnetworks such as G‐0, G‐3, and G‐4 in addition to one of the top central clusters. This strategy would provide a maximum synergistic benefit to an antifibrotic therapy.

In conclusion, this study demonstrates that systems biology and systems pharmacology approaches could be employed to identify and prioritize druggable therapeutic targets that regulate multiple pathologic pathways, while minimizing on‐target toxicity. Even for repurposing approved drugs as well as identifying rational combination therapies, candidate drugs could be tested using this framework to assess their efficacy at the systems biology and pharmacology levels. The framework presented in this study may be instrumental to effectively facilitate the translation of preclinical findings into clinical therapies.

## 4 Experimental Section

### Mice

Thirteen‐week‐old male C57BL/6J mice were obtained from The Jackson Laboratory (Bar Harbor, ME, USA). *Cnr1^−/−^* mice were generated as described.^[^ ^56^ ^]^ *Cnr1^−/−^* (CB_1_R KO) mice were on a C57Bl/6J genetic background. Mice were housed under a 12 h light/dark cycle and fed a standard diet, ad libitum (Teklad NIH‐31; Envigo, Huntingdon, UK). All animal procedures were conducted in accordance with the rules and regulations of the Institutional Animal Care and Use Committee of the National Institutes of Alcohol Abuse and Alcoholism under the protocols of LPS‐GK1.

### Oropharyngeal Aspiration of Bleomycin

A bleomycin‐induced pulmonary fibrosis model was generated by delivering a single dose of bleomycin (1 U kg^−1^) via oropharyngeal aspiration as previously described.^[^ ^29^ ^]^ Briefly, mice were anesthetized with intraperitoneal (ip) injection of Ketamine/Xylazine (80 + 4 mg mL^−1^, respectively, 1 mL kg^−1^) and placed on a surgery board in the supine position at an angle degree of 45°, with the head immobilized by an elastic band across the upper incisors. Using a sterile forceps, the mouth was then nudged open, and the tongue was gently pulled out and toward the mandible and lower incisors. This maneuver allowed visualization of the vocal cords under adequate lighting. Bleomycin was delivered to anesthetized mice through the oropharynx using a sterile 100 µL pipette during inspiration at a volume of 100 µL/50 g body weight. Sterile saline was used as vehicle and applied to the control groups. The animals were then allowed to recover from the anesthesia.

### Pulmonary Function Tests

Respiratory system mechanics measurements were performed using the FlexiVent FX system (SCIREQ Inc., Montreal, Canada) as previously described.^[^ ^57^ ^]^ FlexiVent FX system was equipped with an FX1 module and negative pressure forced expiration extension for mice. FlexiWare v7.2 software was used to operate the system. Forced oscillation techniques and forced expiration measurements were conducted as described previously.^[^ ^58^, ^59^ ^]^ Pulmonary function measurements were performed at the end of the study as a terminal procedure. Mice were anesthetized by intraperitoneal (IP) injection of Ketamine/Xylazine, then an 18‐gauge metal cannula was inserted into the trachea by small incision. Pancuronium was then administered by IP injection (0.8 mg kg^−1^) to induce paralysis before connecting mice to FlexiVent and starting ventilation. Airway resistance, tissue damping (G), tissue elastance (H), and forced expiratory volume per 0.1 s (FEV 0.1), forced vital capacity, Inspiration capacity parameters were measured. Mouse tissue was collected after performing lung function tests.

### Hydroxyproline Measurements by LC‐MS/MS

Pulmonary fibrosis was quantified by measuring hydroxyproline (Hyp) content of left lung using LC‐MS/MS as described previously.^[^ ^60^ ^]^ Briefly, left lung tissue was homogenized in 600 µL of ice‐cold 0.1 n perchloric acid (PCA). One mL 12 n HCl was added to the 600 µL lung homogenate and the homogenate was hydrolyzed at 100 °C for 4 h. Hydrolyzed samples were vortexed and centrifuged at 10 000 g for 10 min, and 5 µL hydrolysate was diluted 200‐fold by the addition of 990 µL of 0.1 n PCA and 5 µL of L‐Proline‐^13^C_5_,^15^N as internal standard. Liquid chromatography tandem mass spectrometry (LC‐MS/MS) analyses were conducted on an Agilent 6470 triple quadrupole mass spectrometer (Agilent Technologies) coupled to an Agilent 1200 LC system. 4‐Hydroxyproline was separated using an Intrada Amino Acid column, 50 × 3 mm, 3 µm (Imtakt) at 40 °C. Mobile phases consisted of acetonitrile/tetrahydrofuran/25 mm ammonium formate/formic acid = 9:75:16:0.3 (v/v/v/v) (phase A) and acetonitrile/100 mm ammonium formate = 20:80 (v/v) (phase B). Gradient elution (600 µL min^−1^) was initiated and held at 0% B for 3 min, followed by a linear increase to 17% B by 6.5 min. This was followed by a step increase to 100% B, which was held until 10 min after the gradient had begun, and then by a linear decrease to 0% B by 11 min, which was held until 13 min after the gradient had begun. The mass spectrometer was set for electrospray ionization operated in positive ion mode. The source parameters were as follows: capillary voltage, 4000 V; gas temperature, 330 °C; and drying gas, 8 L min^−1^. Nitrogen was used as the nebulizing gas. Collision‐induced dissociation (CID) was conducted using nitrogen. Hydroxyproline level was analyzed by multiple reaction monitoring. L‐Proline‐^13^C_5_,^15^N (Sigma, cat#6 08 114) was used as the internal standard. The molecular ion and fragments for hydroxyproline were measured as follows: m/z 132.1→86 and 132.1→68 (CID energy: 8 V and 20 V, respectively). Lung levels of hydroxyproline were determined against a standard curve, using trans‐4‐hydroxy‐L‐proline as standard (Sigma‐Aldrich). Values are expressed as nmol/mg wet tissue

### Masson's Trichrome Staining

The left lung was used for histology. During lung harvesting left lung was excised and freshly frozen by embedding in tissue‐tek optimal cutting temperature (O.C.T.) compound (FisherScientific, USA) on dry ice, and then stored at −80 °C freezer until used. Frozen tissues were sectioned (10 µm) onto glass slides by Cryotome (LEICA 3050S). Histological staining was performed using Masson's Trichrome Kit (Epredia Richard–Allan Scientific) with a slight optimization of the supplier's microwave staining protocol. 10 µm tissue sections were stained with the following time adjustments: DI water rinse adjusted to 5 min after Bouin's Fluid; Weigert's Iron Hematoxylin stain adjusted to 3 min; Biebrich Scarlet‐Acid Fuchsin solution adjusted to 1 min; and Aniline Blue Solution adjusted to 10 min. All other steps were performed as instructed. Histological images were taken by Axio Imager M2 (Zeiss) using ZEN 3.1 (blue edition) software.

### Ashcroft Scoring

Ashcroft scoring was performed as previously described.^[^ ^61^ ^]^ Briefly, images were taken at 20× magnification from at least five randomly selected areas per lung tissue slides. Three readers scored the same fields independently (0 = no fibrosis; 8 = severe fibrosis) and were blinded to study group.

### RNA Extraction and Sequencing

Lower right lobe of the lung tissues was immediately placed in RNAlater solution, then held on ice for 4–5 h, and then stored at −80 °C until the RNA extraction procedure. RNA extraction was performed using RNeasy Mini Kits from Qiagen (Valencia, CA). RNA concentrations were measured with a NanoDrop One (ThermoFisher). One microgram of total RNA was reverse transcribed to cDNA using Bio‐Rad iScript cDNA synthesis kit (Hercules, CA) according to the manufacturer's instructions.

RNA Quality control, library preparations, sequencing reactions, and initial bioinformatic analysis were conducted at GENEWIZ, LLC (South Plainfield, NJ, USA). RNA samples were quantified using Qubit 2.0 Fluorometer (Life Technologies, Carlsbad, CA, USA) and RNA integrity was checked using Agilent TapeStation 4200 (Agilent Technologies, Palo Alto, CA, USA). RNA‐sequencing libraries were prepared using the NEBNext Ultra II RNA Library Prep Kit for Illumina using manufacturer's instructions (NEB, Ipswich, MA, USA). Briefly, mRNAs were first enriched with Oligo(dT) beads. Enriched mRNAs were fragmented for 15 min at 94 °C. First strand and second strand cDNAs were subsequently synthesized. cDNA fragments were end repaired and adenylated at 3’ends, and universal adapters were ligated to cDNA fragments, followed by index addition and library enrichment by limited‐cycle PCR. The sequencing libraries were validated on the Agilent TapeStation (Agilent Technologies, Palo Alto, CA, USA), and quantified by using Qubit 2.0 Fluorometer (Invitrogen, Carlsbad, CA) as well as by quantitative PCR (KAPA Biosystems, Wilmington, MA, USA).

The sequencing libraries were clustered on flowcells. After clustering, the flowcells were loaded on to the Illumina HiSeq instrument (4000 or equivalent) according to manufacturer's instructions. The samples were sequenced using a 2 × 150 bp Paired End configuration. Image analysis and base calling were conducted by the HiSeq Control Software. Raw sequence data (.bcl files) generated from Illumina HiSeq was converted into fastq files and de‐multiplexed using Illumina's bcl2fastq 2.17 software. One mismatch was allowed for index sequence identification.

### Real‐Time PCR Analyses

Expression of the target gene was quantified with gene‐specific primers and PowerSYBRGreen master mix using a QuantStudio 3 Real‐Time PCR instrument from ThermoFisher Scientfic. Predesigned mouse *Tbp* (QT00198443), *Fn1* (QT00135758), *Timp1* (QT00996282), *Vim* (QT00159670), *Col1a* (QT00162204)*, Irf5* (QT00252623), *Irf7* (QT00245266), *Traf4* (QT00325157), *Nfil3* (QT00265104), and *Mef2a* (QT00137480) were purchased from Qiagen (Valencia, CA). The house‐keeping gene TATA‐Box Binding Protein (*Tbp*) was used as the loading control. Gene expression values were calculated based on the ∆∆Ct method.

### Untargeted Metabolomics Profiling

Upper and middle right lung tissues from all samples were sent for untargeted metabolomics profiling to Human Metabolome Technologies, Inc (Boston, MA, USA) using Capillary Electrophoresis Mass Spectrometry (CE‐MS) and Liquid Chromatography Time‐of‐Flight Mass Spectrometry (LC‐TOFMS) analysis. CE‐MS was used to detect polar metabolites whereas LC‐TOFMS was used to detect lipid metabolites.

For CE‐TOFMS analysis, upper right lobe of the lung tissue samples was placed in a homogenization tube, along with zirconia beads (5and 3 mm*φ*). Next, 50% acetonitrile in Milli‐Q water (v/v) containing internal standards (20 µm) was added to the tube, according to the volumes listed in Table 1, after which the sample was completely homogenized at 1500 rpm, 4 °C for 120 s × 5 times, using a beads shaker. Following this, the same amount of 50% acetonitrile in Milli‐Q water (v/v) was added to the mixture, and further homogenization was performed. The homogenate was then centrifuged at 2300 × g, 4 °C for 5 min. Subsequently, the upper aqueous layer was centrifugally filtered at 4 °C through a 5‐kDa cut‐off filter (ULTRAFREE‐MC‐PLHCC, Human Metabolome Technologies, Yamagata, Japan) to remove macromolecules. The filtrate was evaporated to dryness under vacuum and reconstituted in Milli‐Q water for metabolome analysis. The compounds were measured in the Cation and Anion modes of CE‐TOFMS and the samples were diluted for the measurement, to improve analysis qualities of the CE‐MS analysis.

For LC‐TOFMS analysis, middle right lobe of the lung tissue samples was placed in a homogenization tube, along with zirconia beads (5 and 3 mm*φ*). Next, 1% formic acid in acetonitrile (v/v) containing internal standards (10 µm) was added to the tube, and the sample was completely homogenized at 1500 rpm, 4 °C for 120 s, three times, using a beads shaker. Following this, Milli‐Q water was added to the mixture, and further homogenization was performed for another 60 s. The homogenate was then centrifuged at 2300 × g, 4 °C for 5 min, after which the supernatant was transferred to a fresh 1.5 mL microtube. 1% formic acid in acetonitrile (v/v) and Milli‐Q water were then added to the homogenization tube. The homogenization and centrifugation steps were repeated once more, and the supernatant was mixed with the previously collected one. The mixed supernatant was filtered through a 3‐kDa cut‐off filter (NANOCEP 3K OMEGA, PALL Corporation, Michigan, USA) at 9100 × g, 4 °C for 30 min to remove macromolecules, and further filtered through a column (Hybrid SPE Phospholipid 55261‐U, Supelco, Bellefonte, PA, USA) to remove phospholipids. The filtrate was evaporated to dryness under nitrogen and reconstituted in 50% isopropanol in Milli‐Q water (v/v) for metabolome analysis. The compounds were measured in the Positive and Negative modes of LC‐TOFMS and the samples were diluted for the measurement, to improve analysis qualities of the LC‐TOFMS analysis.

Peaks detected in the CE‐TOFMS and LC‐TOFMS analyses were extracted using automatic integration software (MasterHands ver. 2.19.0.2 developed at Keio University, Tokyo, Japan) to obtain peak information, which includes m/z, migration time (MT) in CE, retention time (RT) in LC, and peak area. The peak area was then converted to relative peak area by the following equation.

(1) $$\mathrm{Relative}\;\mathrm{Peak}\;\mathrm{Area}=\frac{\mathrm{Metabolite}\;\mathrm{Peak}\;\mathrm{Area}}{\mathrm{Internal}\;\mathrm{Standard}\;\mathrm{Peak}\;\mathrm{Area}\times \mathrm{Sample}\;\mathrm{Amount}}$$

The peak detection limit was determined based on signal‐noise ratio; S/N = 3.

Putative metabolites were then assigned from HMT's standard library and Known‐Unknown peak library based on m/z and MT or RT. The tolerance was ± 0.5 min in MT and ± 0.3 min in RT, ± 10 ppm†4 (CE‐TOFMS) and ± 25 ppm (LC‐TOFMS) in m/z. If several peaks were assigned the same candidate, the candidate was given the branch number.

(2) $$\mathrm{Mass}\;\mathrm{error}\;\left(\mathrm{ppm}\right)\;=\;\frac{\mathrm{Measured}\;\mathrm{Value}\;-\;\mathrm{Theoritical}\;\mathrm{Value}}{\mathrm{Measured}\;\mathrm{Value}}\times 10^{6}$$

### Transcriptomics Data and Transcription Factor Analysis

The raw FASTQ files from RNA‐sequencing results were quantified using Kallisto^[^ ^62^ ^]^ with an index file generated from the Ensembl mouse reference genome (release 105).^[^ ^63^ ^]^ For human IPF patient RNA‐sequencing data, the raw FASTQ files were downloaded from their respective GEO/SRA repository and processed using similar steps with Ensembl human reference genome (release 105). Kallisto output, both estimated count and TPM (Transcript per kilobase million), were summarized to gene expression by summing up all protein‐coding transcript mapped to each protein‐coding gene based on a mapping file from Ensembl Biomart (https://www.ensembl.org/biomart/martview/).

Data exploration was performed using Principal Component Analysis (PCA) function from the *sklearn* package^[^ ^64^ ^]^ in Python 3.7 using genes with TPM > 5. Differential gene expression analysis was done using DESeq2^[^ ^65^ ^]^ package in R via the Rpy2 package in Python 3.7, with rounded estimated counts from Kallisto as the input. Genes with adjusted *p*‐value < 0.05 was considered as significantly differentially expressed. Functional analysis of the gene trends was done using the *Gseapy* package^[^ ^66^, ^67^ ^]^ in Python 3.7, specifically, the *Enrichr* function.^[^ ^68^, ^69^ ^]^ For transcription factor analysis, the group median of estimated counts was used as input for Dynamic Regulatory Events Miner software.^[^ ^15^ ^]^ The custom configuration options are described in Table S6, Supporting Information and on the code repository page.

For functional analysis of the CB_1_R study cohorts, the fold changes and *p*‐value of DESeq2 analysis were used for functional analysis using the PIANO^[^ ^70^ ^]^ package in R. KEGG pathways mapping to genes were retrieved from the Enrichr website library (https://maayanlab.cloud/Enrichr/). The adjusted *p*‐value cut‐off of 0.05 from Gseapy or the distinct direction panel of PIANO was used to define the significance of a pathway.

### Metabolomics Data Analysis

The quantified metabolites were filtered by removing metabolites with less than two samples per time point. Subsequently, the remaining missing values were imputed using k‐Nearest Neighbors imputer at each time point and subsequently analyzed using the PCA function from the *sklearn* package^[^ ^64^ ^]^ in Python 3.7. Statistical analysis was performed on the filtered data by Student *t*‐test from SciPy module^[^ ^57^ ^]^ and the adjusted *p*‐value was calculated using *multipletest* function from the *statsmodel* package^[^ ^71^ ^]^ in Python 3.7. The functional analysis of differential metabolites trend was done using the *Gseapy* package^[^ ^66^, ^67^ ^]^ in Python 3.7, specifically the *Enrichr* function^[^ ^68^, ^69^ ^]^ with metabolites‐pathways mapping provided by the Human Metabolome Technologies, Inc.

### Co‐Expression Network Analysis

The co‐expression networks were generated by calculating pairwise Spearman correlation rank within each omics type using the *spearmanr* function from SciPy^[^ ^57^ ^]^ and the *p*‐values were adjusted using Benjamini–Hochberg method from the *statsmodel* ^[^ ^71^ ^]^ package in Python 3.7. Correlation results were filtered by removing the pairs with adjusted *p*‐value > 0.05. For the gene co‐expression network (GCN), only the top 25% highest positively correlated pairs were kept for further analysis. Subsequently, the filtered correlation tables were loaded into the *iGraph* package^[^ ^72^ ^]^ in the same environment for the downstream analyses, including degree centrality analysis, community/subnetwork detection with Leiden algorithms,^[^ ^11^ ^]^ and the average clustering coefficient calculation to define the subnetworks’ importance. Functional analyses of the subnetworks were performed using the *Enrichr* function^[^ ^68^, ^69^ ^]^ from the *Gseapy* package^[^ ^66^, ^67^ ^]^ for GCN and hypergeometric test (*hypergeom*) from SciPy for metabolite co‐expression network (MCN). To integrate GCN and MCN, metabolic subsystems mapping for both the genes and metabolites were retrieved from https://metabolicatlas.org/, specifically Mouse‐GEM 1.2.0,^[^ ^73^ ^]^ and used as a mapping file for the functional analysis as described above. A hypergeometric test was also employed to identify the association between GCN subnetworks with gene tracks from the transcription factor analysis and the affected GCN subnetworks after the CB_1_R knockout experiment.

### Other Statistical Analysis

Statistical analysis of pulmonary function test parameters and real‐time PCR results was performed by one‐way ANOVA using SciPy^[^ ^57^ ^]^ module in Python 3.7.

## Conflict of Interest

The authors declare no conflict of interest.

## Author Contributions

M.A. and A.B., contributed equally to this work. Conceptualization, M.A., A.B., and R.C.; data curation, M.A, A.B., and R.C.; formal analysis, M.A, A.B., and R.C; investigation, M.A., A.B., K.M.W., J.K.P., L.P., M.B., and R.C.; methodology, M.A., A.B., K.M.W., J.K.P., L.P., M.B., and R.C.; project administration, R.C.; software, M.A., A.B., and R.C.; supervision, R.C.; visualization, M.A., A.B., and R.C.; writing – original draft, M.A. and R.C.; review & editing, M.A., A.B., K.M.W., J.K.P., L.P., M.B., B.R.G., R.C.; funding acquisition, R.C.

## Supporting information

Supporting Information

Supporting Table 1

Supporting Table 2

Supporting Table 3

Supporting Table 4

Supporting Table 5

Supporting Table 6

Supporting Table 7

## Data Availability

The data that support the findings of this study are available in the supplementary material of this article. RNA sequencing data generated from this study have been deposited and can be accessed through GEO accession number GSE213709 (time series bleomycin) and GSE217814 (CB_1_R KO study). The generated networks and codes used for data curation, analysis, and visualization are available at https://doi.org/10.5281/zenodo.7761929. Results can be explored using an interactive web application at https://niaaa.nih.gov/mouselungfibrosisatlas.
