# Supplementary material for: An Integrative Multiomics Framework for Identification of Therapeutic Targets in Pulmonary Fibrosis
Source: Adv Sci (Weinh). 2023 Apr 10;10(16):2207454. doi: 10.1002/advs.202207454 (PMC10238219; doi:10.1002/advs.202207454)
Supplement: Supplementary file 1 — Supporting Information [file ADVS-10-2207454-s004.pdf]

ADVANCED SCIENCE
Open Access

Supporting Information

for *Adv. Sci.*, DOI 10.1002/advs.202207454

An Integrative Multiomics Framework for Identification of Therapeutic Targets in Pulmonary Fibrosis

*Muhammad Arif, Abhishek Basu, Kaelin M. Wolf, Joshua K. Park, Lenny Pommerolle, Madeline Behee, Bernadette R. Gochuico and Resat Cinar**

Supplementary Information

An integrative multiomics framework for identification of therapeutic targets in pulmonary fibrosis

Muhammad Arif,[1,2#] Abhishek Basu,[1#] Kaelin M. Wolf,[1] Joshua K. Park,[3] Lenny Pommerolle,[1] Madeline Behee,[1] Bernadette R. Gochuico,[4] and Resat Cinar[1]*

[1]Section on Fibrotic Disorders, National Institute on Alcohol Abuse and Alcoholism, National Institutes of Health, Rockville, MD 20852, USA

[2]Laboratory of Cardiovascular Physiology and Tissue Injury, National Institute on Alcohol Abuse and Alcoholism, National Institutes of Health, Rockville, MD 20852, USA

[3]Laboratory of Physiologic Studies, National Institute on Alcohol Abuse and Alcoholism, National Institutes of Health, Rockville, MD 20852, USA

[4]Medical Genetics Branch, National Human Genome Research Institute, National Institutes of Health (NIH), Bethesda, MD, 20892, USA

# Authors equally contributed

*Correspondence: resat.cinar@nih.gov

Supplementary Figures 1-6 Legends

Supplementary Figure 1: qPCR measurement results of fibrosis-related markers.

Supplementary Figure 2: UpSet plot showing the intersection of DEGs from different timepoints compared to control.

Supplementary Figure 3: Metabolic subsystems connecting all GCN subnetworks to the MCN subnetworks

Supplementary Figure 4: % composition of GCN subnetworks in Human IPF cohorts

Supplementary Figure 5: Top five enriched Gene Ontology Biological Processes associated with each gene track from the iDREM analysis

Supplementary Figure 6: qPCR measurement result of *Mef2a* in CB_1R study cohort

Supplementary Tables 1-7 Legends:

Supplementary Table 1: Metadata, Pulmonary Function Tests, RNA-seq Count and TPM, Differential Expression Analysis Results from 7-, 14-, 21-, and 28-days post-bleomycin mice vs control. KEGG

Supplementary Table 2: Gene Co-expression Network (GCN) edges (via github), nodes, and subnetworks information.

Supplementary Table 3: Metabolomics data, the mapping to pathways and HMDB ID, and differential metabolites analysis results.

Supplementary Table 4: Metabolite Correlation Network (MCN) edges, nodes, and subnetworks information.

Supplementary Table 5: Differential Expression Analysis results of the human IPF patients from public data

Supplementary Table 6: iDREM options and results (transcription factors, gene tracks, KEGG, and GO enrichment)

Supplementary Table 7: Metadata, Pulmonary Function Tests, RNA-seq Count and TPM, Differential Expression and Functional Analysis (PIANO) Results from control wild-type and 14 days post-bleomycin (wild-type and CB_1R-KO).

Supplementary Figure 1 (Related to Figure 1)

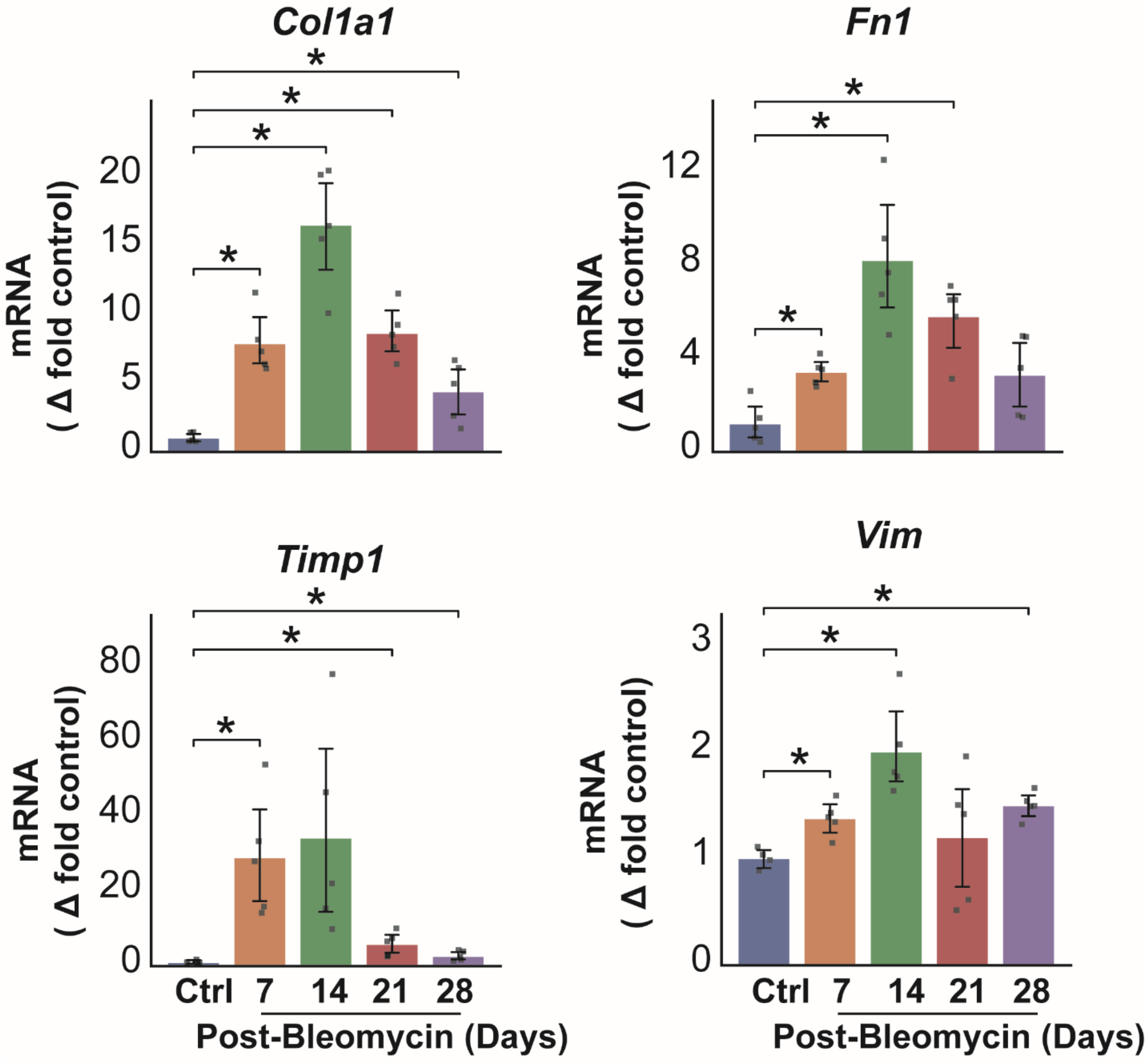

Supplementary Figure 3: qPCR measurement results of fibrosis-related markers (n = 5 per group; related to **Error! Reference source not found.**).

Supplementary Figure 2 (Related to Figure 2)

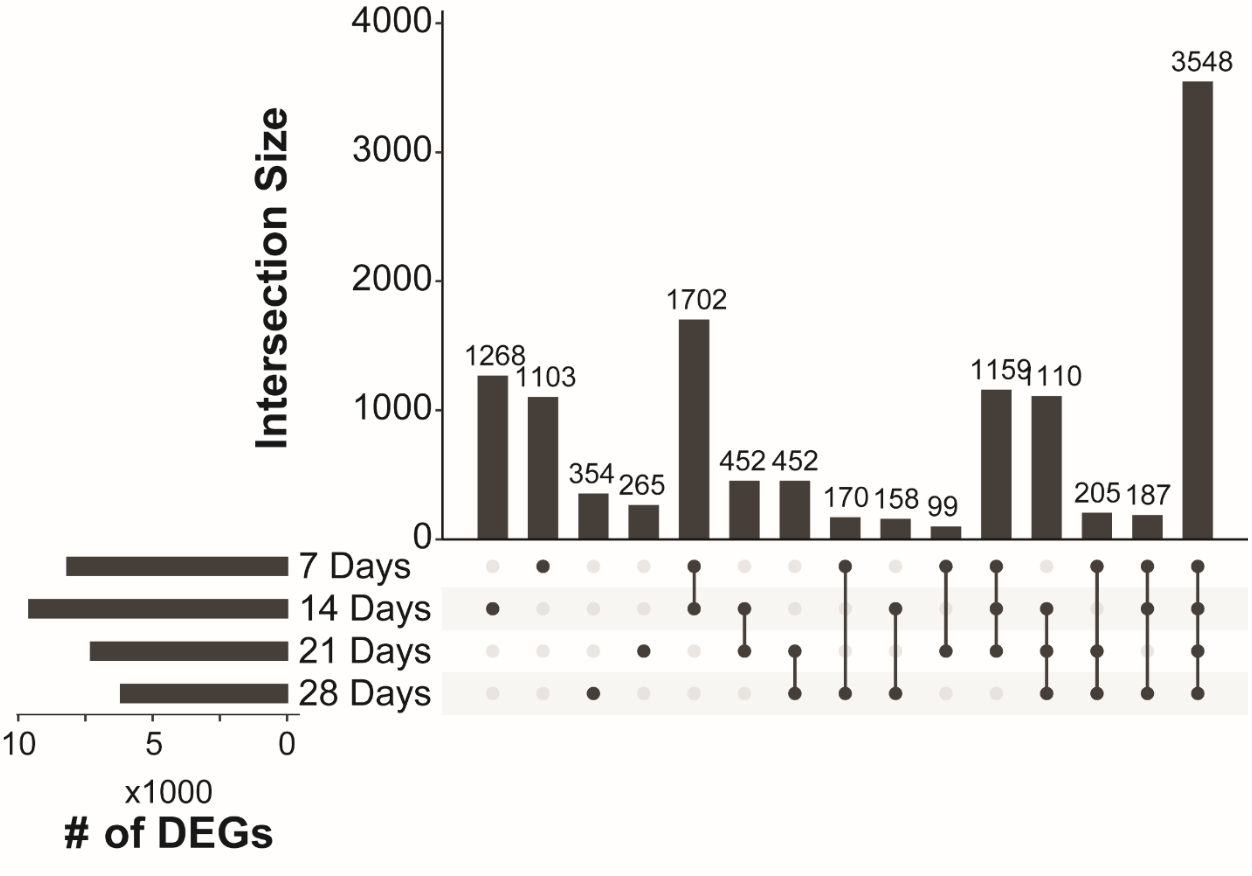

Supplementary Figure 4: UpSet plot showing the intersection of DEGs from different timepoints compared to control where the majority of the DEGs (3548) were commonly dysregulated in all (3548), and 2661 and 3033 DEGs were dysregulated in three and two timepoints, respectively (FDR < 0.05; related to **Error! Reference source not found.**).

Supplementary Figure 3 (Related to Figure 5)

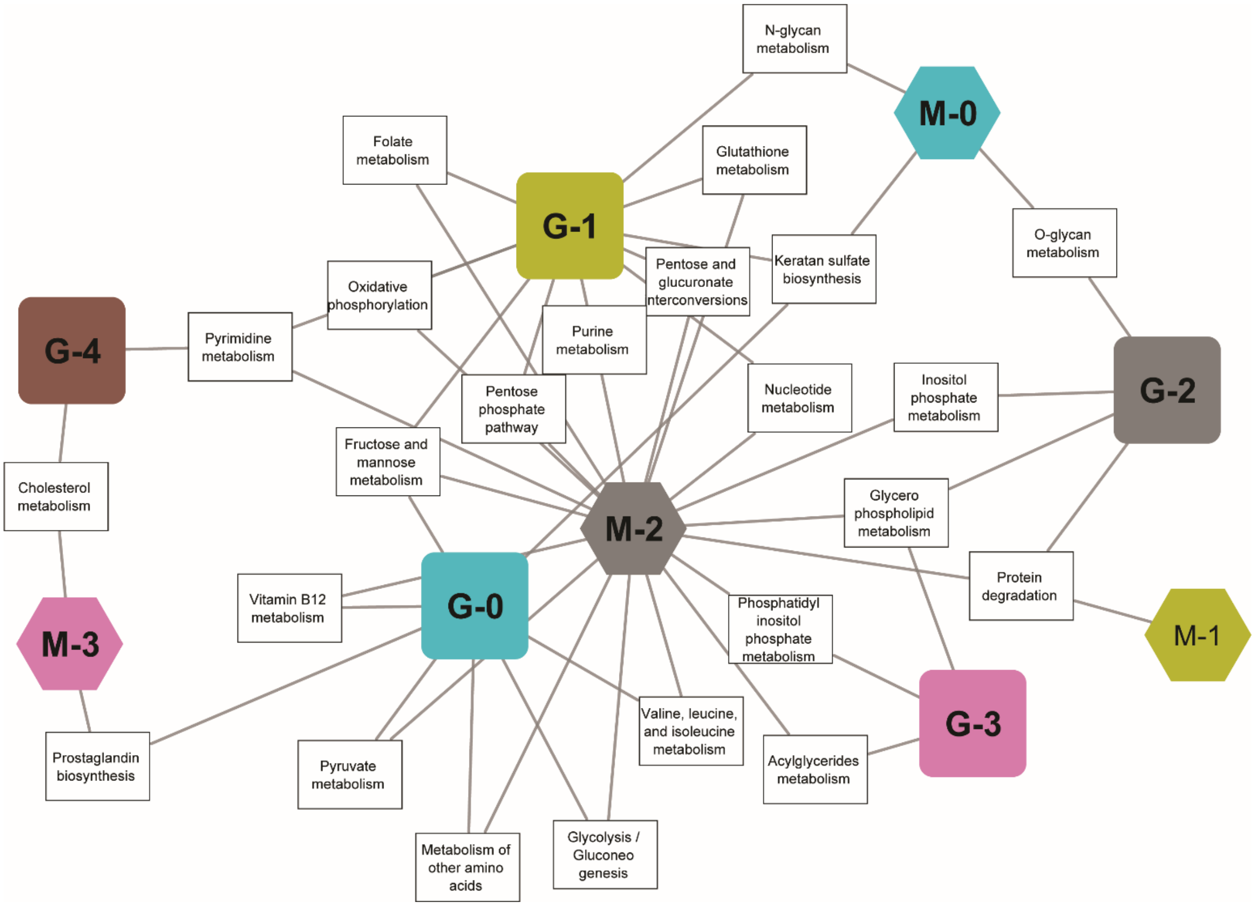

Supplementary Figure 3: Metabolic subsystems connecting all GCN subnetworks to the MCN subnetworks (related to **Error! Reference source not found.**).

Supplementary Figure 4 (Related to Figure 6)

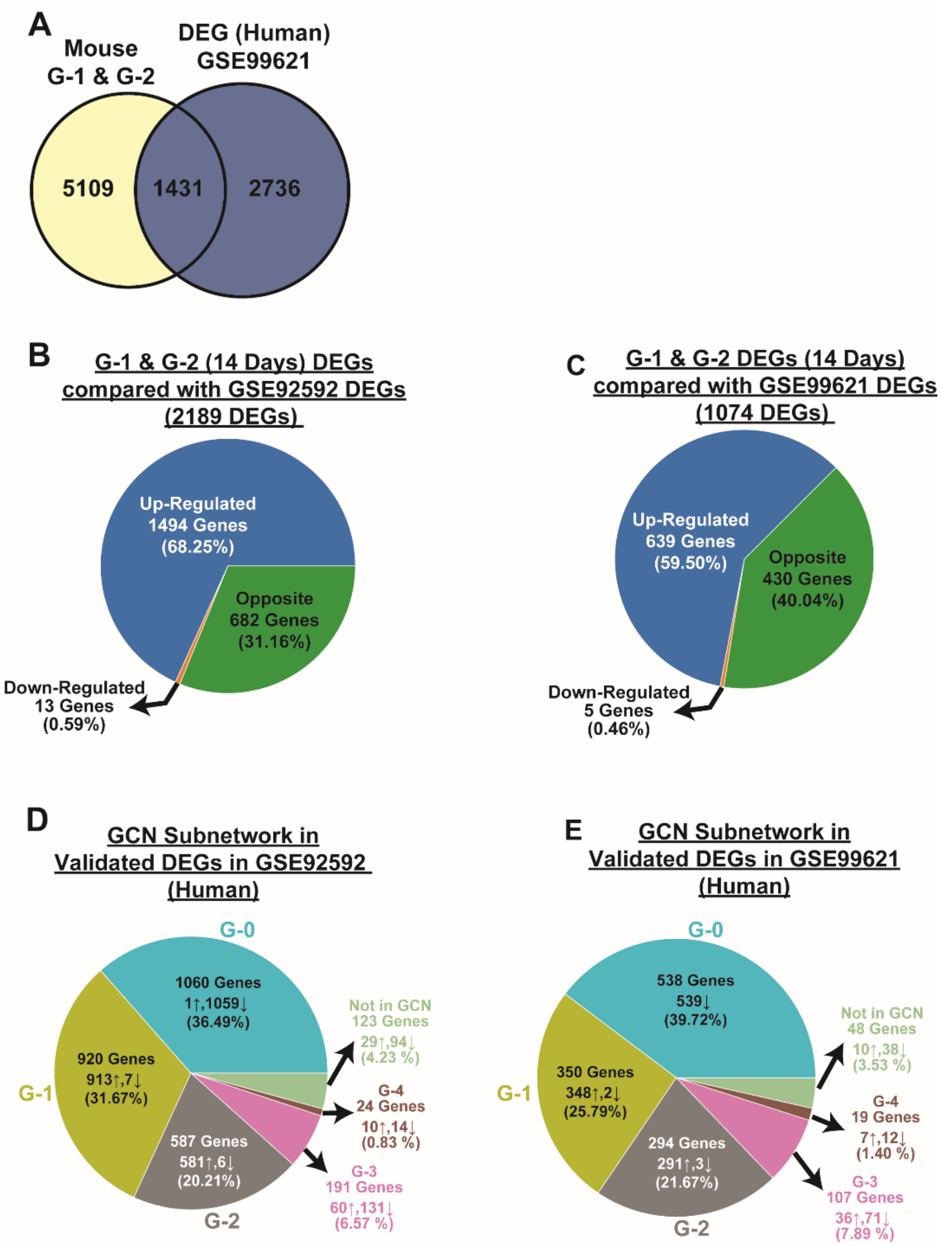

Supplementary Figure 4: (A) 1431 genes from two main GCN clusters (G-1 and G-2) were significantly differentially expressed in late-stage human IPF patients based on an independent cohort (GSE99621). **(B)** 68.84% and **(C)** 59.96% of the intersection DEGs of the two main GCN clusters at 14 Days showed same regulation direction with the late-stage human IPF patients independent cohorts (GSE92592 and GSE99621, respectively). **(D)** GCN composition in Day 14 validated DEGs (mouse) in GSE92592 and **(E)** GSE99621 human IPF cohorts.

Supplementary Figure 5 (Related to Figure 7)

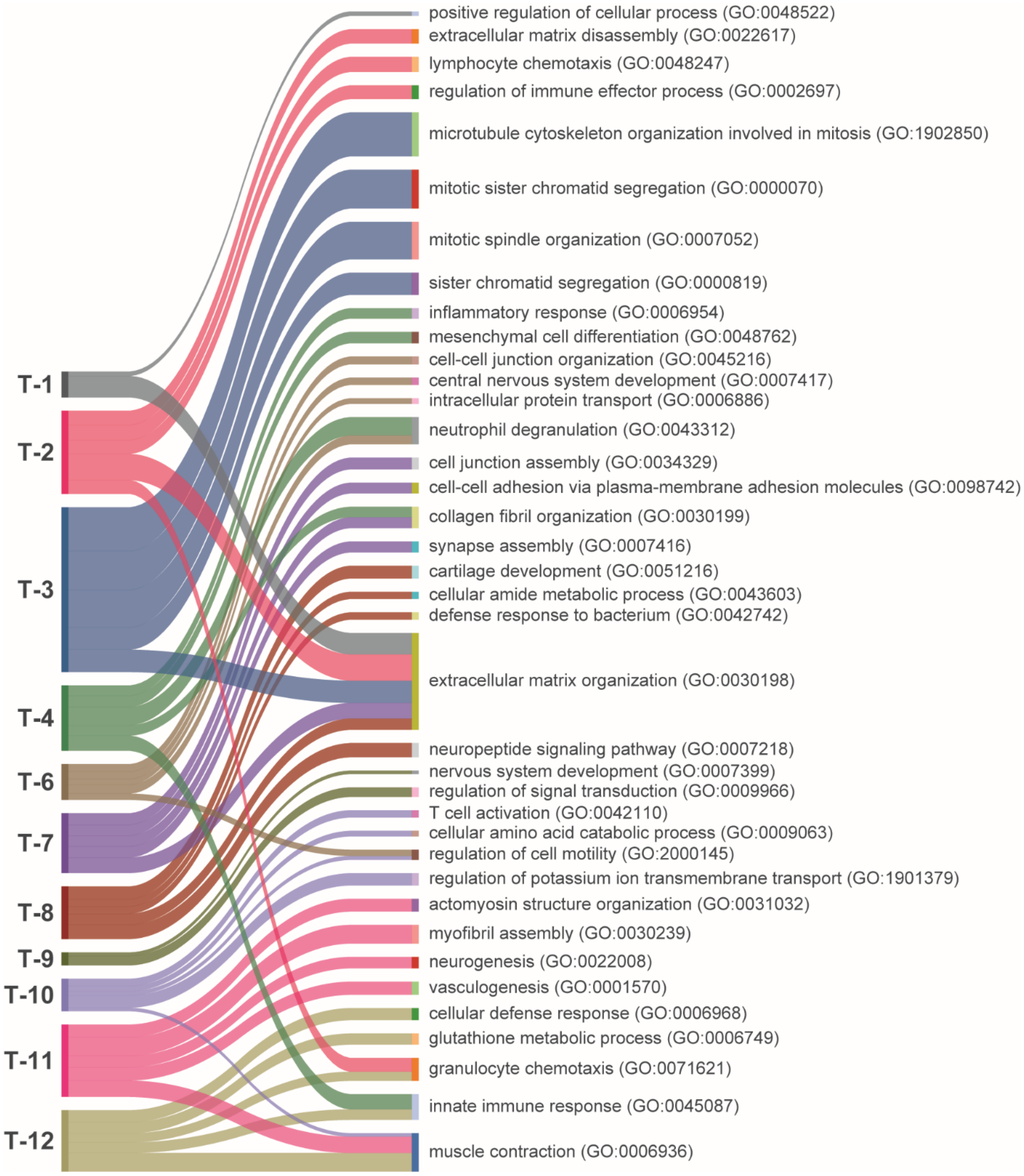

Supplementary Figure 5: Top five enriched Gene Ontology Biological Processes associated with each gene track from the iDREM analysis (related to **Error! Reference source not found.**).

Supplementary Figure 6 (Related to Figure 8)

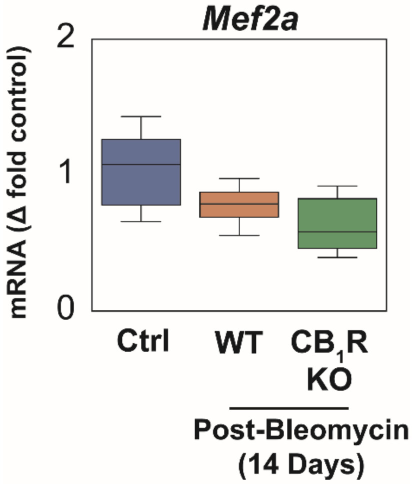

Supplementary Figure 6: qPCR measurement result of *Mef2a* in CB_1R study cohort (related to **Error! Reference source not found.**).
